# Spike encoding techniques for IoT time-varying signals benchmarked on a neuromorphic classification task

**DOI:** 10.3389/fnins.2022.999029

**Published:** 2022-12-21

**Authors:** Evelina Forno, Vittorio Fra, Riccardo Pignari, Enrico Macii, Gianvito Urgese

**Affiliations:** Politecnico di Torino, Electronic Design Automation (EDA) Group, Turin, Italy

**Keywords:** neuromorphic encoding techniques, neuromorphic computing, time-varing signals, spatio-temporal pattern recognition, event based encoding, Spiking Neural Network, benchmarking, IoT applications

## Abstract

Spiking Neural Networks (SNNs), known for their potential to enable low energy consumption and computational cost, can bring significant advantages to the realm of embedded machine learning for edge applications. However, input coming from standard digital sensors must be encoded into spike trains before it can be elaborated with neuromorphic computing technologies. We present here a detailed comparison of available spike encoding techniques for the translation of time-varying signals into the event-based signal domain, tested on two different datasets both acquired through commercially available digital devices: the Free Spoken Digit dataset (FSD), consisting of 8-kHz audio files, and the WISDM dataset, composed of 20-Hz recordings of human activity through mobile and wearable inertial sensors. We propose a complete pipeline to benchmark these encoding techniques by performing time-dependent signal classification through a Spiking Convolutional Neural Network (sCNN), including a signal preprocessing step consisting of a bank of filters inspired by the human cochlea, feature extraction by production of a sonogram, transfer learning *via* an equivalent ANN, and model compression schemes aimed at resource optimization. The resulting performance comparison and analysis provides a powerful practical tool, empowering developers to select the most suitable coding method based on the type of data and the desired processing algorithms, and further expands the applicability of neuromorphic computational paradigms to embedded sensor systems widely employed in the IoT and industrial domains.

## 1. Introduction

In recent years, technology improvements have made embedded devices more powerful and more accessible than ever before (Dahlqvist et al., 2019). This has enabled significant growth in the use of Machine Learning (ML) techniques in Internet of Things (IoT) and edge computing applications featuring such devices (Branco et al., 2019). Deploying ML models directly on the remote device, rather than providing them as a service through the cloud, has a series of advantages. First of all, edge devices can provide real-time inference, reducing the latency of the system in timing-critical tasks such as autonomous driving. Secondly, keeping all data on the device makes the application more reliable, avoiding the repercussions of network shortages; at the same time, as data is not transmitted, user privacy is improved. Finally, the elimination of the cloud dependency reduces the power consumption from communication functions as well as the cost of the overall infrastructure^1^.

While the production of ML code for embedded devices has been significantly facilitated over the years, it has retained a few challenges. Since edge devices remain constrained in terms of power, memory and computational resources (Loyez et al., 2021), the candidate models must be carefully chosen not only based on the type of input data, but also on the hardware's requirements. After acquiring a large amount of sample data through the same embedded sensors that will be used in the final application, the models undergo a training phase: this phase is usually executed on a server machine due to its high computational demand. Several models can be assessed for performance, and the most promising is selected for deployment on the machine. Dedicated end-to-end solutions for data acquisition, labeling, model optimization and deployment are provided through major hardware manufacturers (e.g., Qeexo AutoML^2^.) as well as in open source releases (such as the Embedded Learning Library^3^).

Without a doubt, the area of ML that has received the most attention by the scientific and industrial communities during the past decade is that of Artificial Neural Networks (ANN). Most ANN models are too resource intensive to run on embedded hardware, requiring high amounts of memory and dedicated, power-hungry devices such as GPUs. A novel type of ANN that fulfills the edge computing requirements of low power consumption, localized memory and real-time response is the Spiking Neural Network (SNN), a biologically-inspired model based on the behavior of animal neurons and characterized by sparse computation (Ghosh-Dastidar and Adeli, 2009). This type of network can be accelerated on compact, low-power neuromorphic hardware (such as SpiNNaker Furber et al., 2012, Intel Loihi Davies et al., 2018, and Dynap-SEL Moradi et al., 2017), and exhibits the potential to implement both offline and online learning (Tavanaei et al., 2019), though a lot of work remains to be done on the software support (Rhodes et al., 2018; Knight et al., 2021) and compilers (Urgese et al., 2015, 2016; Lin et al., 2018) in order to attain said goal.

The most suitable configurations of data encoding, network type, training method, and hardware platforms allowing to exploit the advantages of SNNs are still subject to ongoing research. Yet, despite still being an emergent technology, various neuromorphic applications are beginning to surface in the field of embedded systems (Schuman et al., 2022). Using standard industry tools, neuromorphic hardware can already be seamlessly integrated with an embedded processor on the same chip, providing SNN-based co-processing (Forno et al., 2021b). Areas of implementation explored in literature include image and video frame analysis (Abeysekara and Abdi, 2019), dataset clustering (Bako, 2009), pedestrian detection (Lee and Park, 2019; Kang et al., 2020), self-driving robots (Hwu et al., 2017) and robotic fine-touch sensing including dynamic motor control and Braille (Bologna et al., 2013) or texture recognition (Friedl et al., 2016); neuromorphic computing platforms can also be paired with natively event-driven sensors for greater efficiency, such as DVS cameras for gesture recognition (Massa et al., 2020) or robotic vision (Bartolozzi et al., 2011). There is also growing interest in neuromorphic applications as suitable candidates for the implementation of human-related time series data analysis: while deep learning techniques have found success in the classification of time-variant data, their implementation on resource-bound hardware runs into issues related to the need for signal pre-processing as well as for identification of long and short dependencies within the data, which influences the efficiency of the chosen model (Christensen et al., 2022). In previous work by the authors (Fra et al., 2022), SNN-based solutions compared favorably to ANN implementations, while demonstrating a reduction in energy consumption.

In order to be processed by a SNN, input analog and digital data must be converted into a stream of spiking signals. However, biological research shows that even in nature, sensory data can be translated into spikes in various different ways; again taking inspiration from the animal neuron, a number of encoding schemes have been derived (Auge et al., 2021). *Rate-based encodings* have been used since the early days of SNN research (Brader et al., 2007), and have since shown to attain the greatest efficiency when used for conversion of trained ANNs into SNNs (Diehl et al., 2015; Esser et al., 2015; Rueckauer et al., 2017) for classification applications. In the realm of neuromorphic sensors, an event-based cochlea (Liu et al., 2010) transcodes audible frequency amplitudes into neuron firing rates; rate codes are also popular in the area of robotic control (Bing et al., 2018). By contrast, research into *Temporal Coding* schemes has received new interest in recent years. While these techniques have also been employed in converted networks (Kim et al., 2018; Rueckauer and Liu, 2018; Zhang et al., 2019), resulting in power savings, a variety of applications has emerged that uses time-based encodings natively. These include bio-inspired olfactory sensors (Chen et al., 2011) and cameras (Delbrück et al., 2010), hybrid ANN/SNN (Liu and Yue, 2017) and fully spiking (Kheradpisheh et al., 2018; Park et al., 2019; Sboev et al., 2020) networks for image classification, speaker authentication (Wysoski et al., 2007) and speech recognition systems (Loiselle et al., 2005; Schrauwen et al., 2008), time series forecasting (Sharma and Srinivasan, 2010) and anomaly detection (Ahmad et al., 2017; Chen and Qiu, 2017), and many more.

In this paper, we aim to analyze the impact of different spike encoding techniques on a spiking Convolutional Neural Network trained by the process of transfer learning. The resulting networks are tested on time-varying input signals, originally acquired from digital sensors and encoded into spikes before being fed to the classifiers. While encoding digital data does not guarantee as good a result as operating directly with spike-domain sensors, we adopt this approach because of the abundance of digital-output sensors available on the market, compared to the scarcity of neuromorphic sensors; this also ensures fairness in the comparison between various encodings, as they are all produced from the same input data. Thus, the analysis presented here also represents a guideline for future development of algorithms and encoding/decoding techniques for integrated System of Systems featuring interoperability of various modules with neuromorphic sensing solutions.

## 2. Materials and methods

**Figure 1:**
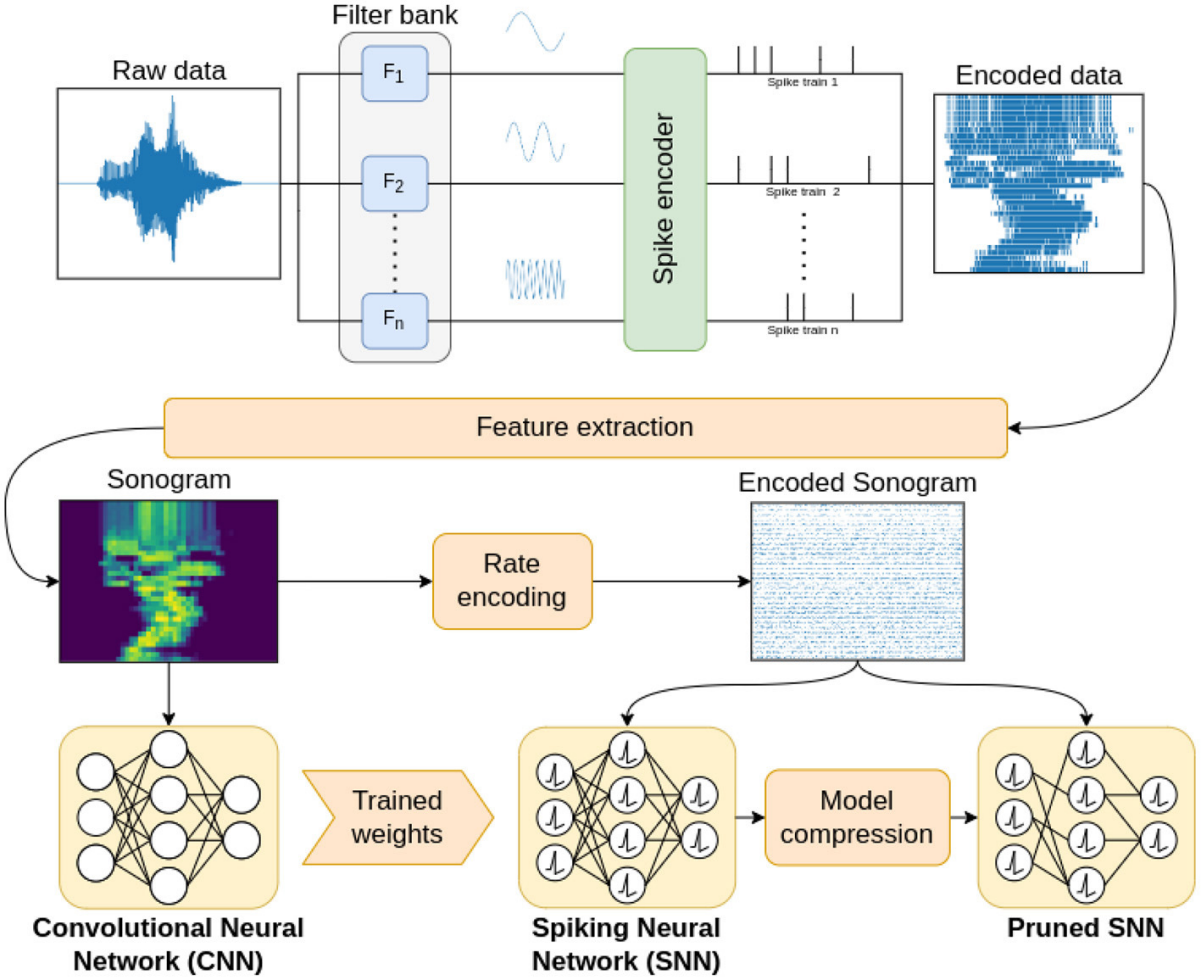
Block diagram of the proposed encoding benchmarking pipeline, including a frequency decomposition step through a filter bank, a spike encoding step, feature extraction by production of the sonogram, transfer learning with a non-spiking network, and model compression.

In this work, we investigated the impact of input signal encoding when employing transfer learning to train a spiking convolutional neural network (sCNN). The choice of a convolutional network architecture is common practice for time-varying signals (Dominguez-Morales et al., 2018; Sharan et al., 2020; Rashid et al., 2022) as it allows to sidestep the use of recurrent neural networks, which are more complex and computationally intensive (Fra et al., 2022). Figure 1 summarizes the pipeline used to conduct this investigation. The *filter bank* block aims to reproduce the behavior of the human cochlea through the use of an array of filters that decomposes raw data into different frequency channels. Different types of algorithms belonging to the *rate-based* and *temporal coding* families are then used to encode the data, translating it into the spike domain. Through the *feature extraction* process we produce the *sonogram*, a representation of the encoded spike-domain signal in the form of an image by means of a time-binning process. The sonogram is used to perform the transfer learning method, enabling us to indirectly train an SNN network through learning techniques employed in the ANN field. Finally, to verify the accuracy performance, the sonogram is re-encoded into the spike domain. Different CNN/SNN configurations are tested in order to achieve the best classification accuracy. A model compression step is applied to the SNN to reduce its dimensions by means of the progressive elimination of synaptic connections between neurons based on their weight.

### 2.1. Dataset

We selected time-varying signals from two different datasets. Their distinction can be made from a twofold perspective: on the one hand, the type of activity involved, on the other hand, the signal frequency. While the former can be straightforward and it is easily related to the nature of the data, much more than to the specific models and methods employed, the latter is more inherently linked to the neuro-inspired pre-processing steps performed. The two datasets are the Free Spoken Digit (FSD) Dataset^4^, providing audio signals, and one of the datasets typically employed for human activity recognition (HAR) (Fra et al., 2022), namely the WISDM Smartphone and Smartwatch Activity and Biometrics Dataset (Weiss, 2019; Weiss et al., 2019). Beside the distinction based on the type of activities the two datasets are collected from, the one referring to the signal frequency can be made based on the human audible spectrum: very low frequency below 20 Hz, low frequency from 20 to 500 Hz, middle frequency from 500 to 2 kHz and high frequency from 2 to 20 kHz. Based on these definitions and the Nyquist-Shannon sampling theorem, the samples provided by the FSD dataset and the WISDM dataset can be assigned to the middle frequency and to the very low frequency range respectively.

The Free Spoken Digit (FSD) Dataset is a collection of audio signals acquired with a frequency of 8 kHz. In its latest version, each spoken digit is recorded 50 times from 6 speakers with English pronunciation but different accents. All the samples are trimmed, so that similar silence intervals are present both at the beginning and at the end. This dataset has been previously used in works investigating spike encoding in the neuromorphic domain (Peterson, 2021).

The WISDM Smartphone and Smartwatch Activity and Biometrics Dataset was published in 2019 by the Wireless Sensor Data Mining (WISDM) Lab. Based on 3D data acquired through accelerometer and gyroscope from smartphone and smartwatch, it collects signals related to 18 different activities performed by 51 subjects, with an acquisition rate of 20 Hz and a total duration of 3 min for each activity. Differently from the previous version of the WISDM dataset (Kwapisz et al., 2011), it also ensures a significant class balance, with a relative contribution of each activity ranging from 5.3 to 5.8% of the 15,630,426 total samples.

**Figure 2:**
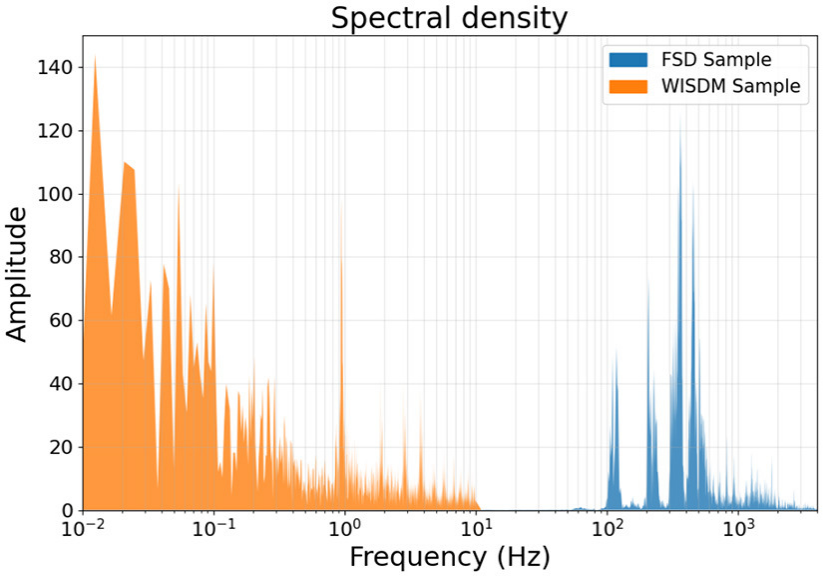
Spectral densities of a sample from the FSD and one from the WISDM dataset. The accelerometer-acquired WISDM data belongs to the very-low frequency range, while the FSD sample belongs to the middle range, as is typical for human-audible signals.

In Figure 2, the spectral density for both datasets are represented, showing how the two types of data occupy different parts of the frequency spectrum with no overlap.

### 2.2. Pre-processing

As is well known, biology and the animal kingdom represent an incredibly rich source of ideas for human development. In this work, we drew inspiration from them to implement some pre-processing techniques for the time-varying signal we took into account. Specifically, adopting a methodology inspired by the human auditory system, we implemented a procedure mimicking the working principle of the cochlea. This latter represents the terminal part of the auditory apparatus, and it consists of a spiral structure whose nerve cells, thanks to their arrangement along the so-called basilar membrane, allow a behavior similar to that of a filter-bank. As a result, a frequency decomposition of the incoming stimulus is performed, and each of the resulting components, identified through the excitation of specific regions of the basilar membrane with matching characteristic frequency, is translated into pulses producing the electrical signal to be treated by the brain (Greenwood, 1961; Hachmeister, 2003; Gomez and Stoop, 2014; Oxenham, 2018; Schurzig et al., 2021). In literature, it has been shown that gammatone and Butterworth filters are suitable solutions to reproduce such a mechanism (Johannesma, 1972; Katsiamis et al., 2006; Zhang and Abdulla, 2006; Elias and George, 2014; Sharan et al., 2020); moreover, by adequately adjusting the frequency range, this type of filter bank can also perform effective feature extraction on other time-varying signals, such as vibrations recorded by an accelerometer (Dennler et al., 2021). In this work, we applied them to perform a pre-processing step on the considered data, resulting in a split of the time-varying signals into different frequency channels. Details about their definition can be found in the Supplementary material.

### 2.3. Encoding techniques

Although the availability of neuromorphic, event-based sensors is increasing, as demonstrated by the commercialization of silicon retina cameras by Sony^5^ and Prophesee (Blackman, 2019), spiking neural networks are typically employed for the analysis of continuous data coming from conventional sensors. As a consequence, a spike encoding of these signals is needed in order to produce sparse, event-based input data. Two possible approaches exist for spike generation: on the one hand, similarly to what is defined as the Representation Principle of the Neural Engineering Framework (NEF) (Eliasmith and Anderson, 2003), the neural response to a continuous signal is produced relying on specific neuron models and characteristics; on the other hand, a continuous signal is transformed into discrete spikes by means of a number of possible algorithms. In this work, we focus on the latter, which ensures, in the framework of IoT applications, the possibility of fully exploiting neuro-inspired strategies even in the absence of dedicated neuromorphic hardware.

**Figure 3:**
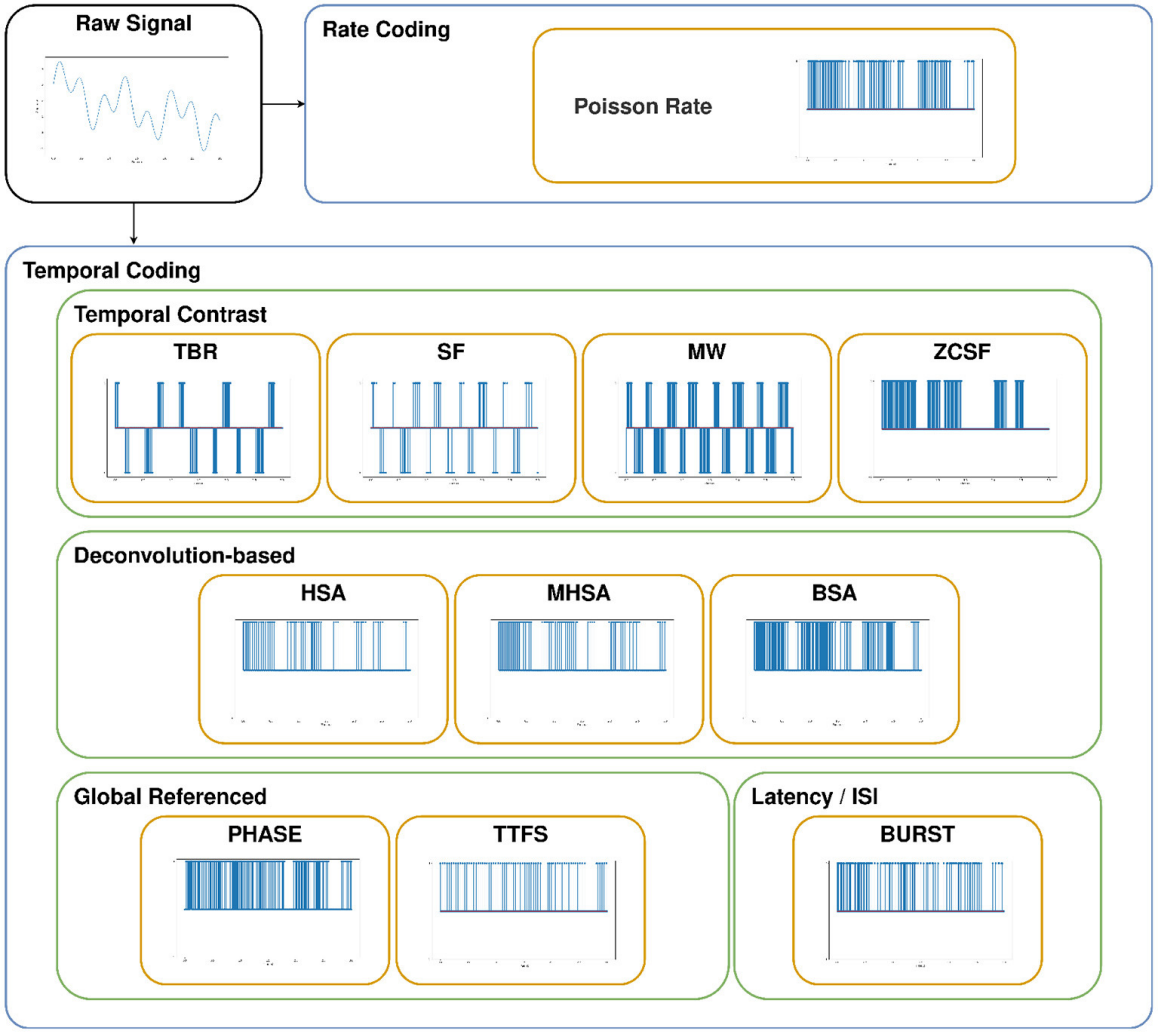
Example of spike trains produced by each of the adopted encoding techniques, given an arbitrary input signal.

Encoding algorithms for spike generation can be classified according to two main categories, Rate Coding and Temporal Coding, which present significant differences in the number of degrees of freedom allowed in the encoding: in Rate Coding a signal is encoded by the number of spikes per time unit, while Temporal Coding comprises a variety of approaches. For all the hereinafter discussed and adopted techniques, pseudocode is reported in the Supplementary material and an example of spike train generation is shown in Figure 3. In the following, we will provide the details of the encoding techniques used in the study, each belonging to one of the two aforementioned coding categories.

#### 2.3.1. Rate coding

Widely adopted for ANNs due to its ease and robustness, rate coding employs a mechanism for information representation based on the number of spikes per unit time (Guo et al., 2021).

##### 2.3.1.1. Poisson Rate

Among the different algorithms belonging to the class of rate coding (Auge et al., 2021), we employed the one adopting the Poisson distribution to generate spike trains. Specifically, by means of this mechanism, the probability of having *n*∈ℕ spikes in a time interval Δ*t* is:


(1) $$\begin{array}{l} P_{n}\left(\Delta t\right)=\frac{{\left(r\Delta t\right)}^{n}}{n!}e^{-r\Delta t} \end{array}$$

Where *r*∈ℝ, referred to as the spike rate, is the real value to be encoded.

From an operative standpoint, the implementation of this algorithm can be performed through the following steps (Liu et al., 2016):

1. Definition of the time interval Δ*t* in which to generate the spike train;
2. Generation of a sequence of random numbers *x*∈[0, 1]⊂ℝ;
3. From *t* = 0, definition of spike times *t*_*i*_ as:
  (2) $$\begin{array}{l} t_{i}=t_{i-1}+ISI_{i}\;\text{for}i\geq 1 \end{array}$$
  where
  (3) $$\begin{array}{l} ISI_{i}=\frac{-\log\left(1-x_{i}\right)}{r} \end{array}$$
  is the *i*^th^ inter-spike interval defined as the *i*^th^ time interval in which the probability of having *n* = 0 spikes is equal to *x*_*i*_;
4. Generation of a spike at each time *t*_*i*_ until *t*_*i*_>Δ*t*.

#### 2.3.2. Temporal coding

As already mentioned, temporal coding encloses encoding mechanisms whose information representation strategy relies on multiple features. Besides referring to the number of spikes in a unit time, a distinguishing feature of temporal coding is the possibility of accounting for the exact spike timing to carry information (Dupeyroux et al., 2021). Additionally, characteristics like the relative spike timing and the temporal spacing between spikes can be exploited too. Depending on which of these properties is taken into account, five categories of temporal coding algorithms can be identified: Temporal Contrast, Deconvolution-based, Global Referenced, Latency/ISI, and Correlation and Synchrony (Auge et al., 2021).

##### 2.3.2.1. Temporal Contrast

The algorithms belonging to this category mainly focus on the signal variations in time, and they are employed to produce spikes with either positive or negative sign. Because time-based variation is the main feature encoded by this class, it is not well suited for purely spatial data such as still images. Examples of use can be found for audio signals (Liu et al., 2014), electromiography data (Donati et al., 2019), speech recognition (Gao et al., 2019), failure prediction based on machine vibrations (Dennler et al., 2021), and robotic Braille reading (Müller-Cleve et al., 2022).

###### 2.3.2.1.1. Threshold-based representation

The Threshold-Based Representation (TBR) algorithm can be somehow identified as the constitutive member of the Temporal Contrast category (Delbruck and Lichtsteiner, 2007). It encodes information by generating spikes according to the absolute signal variation with respect to a fixed threshold. Specifically, the main steps performed when adopting this technique are:

1. Given a signal composed of *n* channels, variations along each channel are evaluated between consecutive timesteps;
2. For each channel, a specific threshold is defined as:
  (4) $$\begin{array}{l} Threshold=mean\left(Variation\right)+\gamma \cdot std\left(Variation\right) \end{array}$$
  Where γ is a tunable parameter directly reflecting on the amplitude of the noise-reduction band affecting the *Variation* values between −*Threshold* and +*Threshold*. The greater γ, the wider the threshold band, the smaller the number of spikes. Depending on the noise level to be filtered out, different ranges of values for γ can be identified:
  - γ = 0: all the signal variations are kept and the threshold is defined as their mean value;
  - 0 < γ ≤ 1: noise is not a major concern within the signal but small variations are not needed to preserve the information content;
  - γ>1: a relevant noise is present and its impact has to be mitigated when generating spikes.
3. Timesteps for the spike train are defined dividing the Δ*t* interval in which to generate the spikes by the length (*L*) of the input signal;
4. At each timestep, if *Variation* exceeds *Threshold* in absolute value, a spike is emitted with polarity defined by the sign of both *Variation* and *Threshold*.

###### 2.3.2.1.2. Moving window

As for the TBR algorithm, the same underlying idea of using a threshold value is employed in the case of Moving Window (MW). However, differently from the previous encoding strategy, such threshold is employed together with a value referred to as *Base* and defined as the mean of the signal, along each channel, within a sliding window of fixed length:


(5) $$\begin{array}{l} Threshold=mean\left(Variation\right) \end{array}$$

(6) $$\begin{array}{l} Base=mean\left(Signal\left[1:Window\right]\right) \end{array}$$

Furthermore, on the contrary with respect to TBR, the condition for spike emission is verified relying on the signal itself rather than on its variation. When the signal exceeds the value *Base*+*Threshold*, a positive spike is generated, while for signal values smaller than *Base*−*Threshold* a negative spike is produced. Such mechanism for spike generation, following the adoption of a sliding window along the signal, also turns out to be more robust to noise with respect to TBR (Kasabov et al., 2016).

###### 2.3.2.1.3. Step-forward

Proposed by Kasabov et al. (2016) as an improvement with respect to the encoding adopted for the artificial silicon retina in Delbruck and Lichtsteiner (2007), the Step-Forward (SF) algorithm also relies on the idea of an iteratively updated baseline value. Similarly to the case of MW, *Base* and *Threshold* are employed to compute such baseline, and their definitions, for each signal channel, are:


(7) $$\begin{array}{l} Threshold=mean\left(Jump\right)/\gamma \end{array}$$

(8) $$\begin{array}{l} Base=Signal\left[1\right] \end{array}$$

Where *Jump* refers to an array containing the maximum-to-minimum differences for each channel and γ is a tunable parameter. As for TBR and MW, both positive and negative spikes can be produced. Specifically, the former occur when the signal overcomes the value *Base*+*Threshold* while the latter are emitted for signal values lower than *Base*−*Threshold*.

###### 2.3.2.1.4. Zero-crossing step-forward

An alternative implementation of SF is obtained taking advantage of zero-crossings (Wiren and Stubbs, 1956; Kedem, 1986). The resulting Zero-Crossing Step-Forward (ZCSF) algorithm inherits the definition of *Threshold* but does not involve the *Base* value, which is replaced by a half-wave rectifying behavior introduced through the condition *Signal* > 0. With ZCSF, spike emission hence occurs for positive signal values higher than *Threshold*, resulting, differently from the previous encoding schemes, in positive spikes only.

##### 2.3.2.2. Deconvolution-based

Composed of the Hough Spiker Algorithm (HSA) (Hough et al., 1999) and the subsequent modified-HSA and Ben's Spiker Algorithm (BSA) (Schrauwen and Van Campenhout, 2003), this class of encoding techniques originates from the inverse problem of reconstructing an analog signal from a spike train by means of a finite impulse response filter (FIR). Specifically, by reversing such operation, the algorithms belonging to this class provide analog-to-spike conversion employing the convolution function in a subtractive procedure (Hough et al., 1999). As in the case of ZCSF, unipolar spikes are produced.

###### 2.3.2.2.1. Hough spiker algorithm

The progressive subtraction is performed by the HSA by first comparing the value of the analog signal to the result of a given convolution operation. If the signal to be encoded overcomes this latter, it undergoes the subtraction of the convolution value. As a consequence, the distinguishing iterative step in the Hough Spiker Algorithm is, for each signal channel:


(9) $$\begin{array}{l} Signal\left[i+j-1\right]=Signal\left[i+j-1\right]-filter\left[j\right] \end{array}$$

Where *i* identifies the time steps of the signal to be encoded and *filter* is the convolution result, with *j* representing its value indices. In our analysis, we adopted a rectangular window as the convolution function.

###### 2.3.2.2.2. Modified hough spiker algorithm

The Modified HSA, while maintaining the core idea of a subractive, deconvolution-based procedure, differs from the HSA by the adoption of a *Threshold* value. The same operation as in Equation (9) is performed if *error* ≤ *Threshold*, where *error* results from an accumulation, occurring when the input signal does not overcome the convolution function, defined, for each signal channel, as:


(10) $$\begin{array}{l} error=error+\left(filter\left[j\right]-Signal\left[i+j-1\right]\right) \end{array}$$

###### 2.3.2.2.3. Ben's spiker algorithm

In comparison with the previous technique, by the Ben's Spiker Algorithm two cumulative error metrics are introduced, for each signal channel, beside the *Threshold* value:


(11) $$\begin{array}{l} error1=error1+abs\left(Signal\left[i+j-1\right]-filter\left[j\right]\right) \end{array}$$

(12) $$\begin{array}{l} error2=error2+abs\left(Signal\left[i+j-1\right]\right) \end{array}$$

In the original work presenting BSA (Schrauwen and Van Campenhout, 2003), the condition to be checked before applying Equation (9) is that *error*1 does not overcome the value *error*2−*Threshold*. In this work, however, we refer to the implementation proposed in Petro et al. (2020), where the condition is modified as:


(13) $$\begin{array}{l} error1\leq error2\cdot Threshold \end{array}$$

##### 2.3.2.3. Global Referenced

This third class of algorithms for temporal coding encloses techniques whose spike generation mechanism relies on some global temporal characteristic of the input signal. In the case of Phase Encoding, such feature is the time difference with respect to an oscillatory reference (Hopfield, 1995); while Time-to-First-Spike (TTFS) employs the time since the onset of the stimulus (Thorpe and Gautrais, 1998; Johansson and Birznieks, 2004). Similarly to the Deconvolution-based algorithms, both Phase Enconding and TTFS produce spikes with single polarity.

###### 2.3.2.3.1. Phase encoding

The possibility of successfully developing an encoding scheme relying on a phase evaluation with respect to an oscillatory reference was presented in Montemurro et al. (2008). In our work, we refer to the implementation proposed in Kim et al. (2018): the binary representation of the input by β fractional bits is adopted as the oscillatory reference, after rectifying and normalizing the signal, for each channel, into the range [0, 1].

###### 2.3.2.3.2. Time-to-first-spike

In Rueckauer and Liu (2018), different strategies to apply Time-to-First-Spike encoding have been investigated, depending on the threshold definition for the membrane potential. In our work, we implement a dynamic threshold by means of an exponentially decaying function as in Park et al. (2020):


(14) $$\begin{array}{l} P_{th}\left(t\right)=\theta_{0}e^{-t/\tau_{th}} \end{array}$$

where θ_0_ is a constant and τ_*th*_ represents the decay time of the membrane potential. For the here reported investigation, we used θ_0_ = 1 and τ_*th*_ = 0.1. With respect to other implementations, we also adopted a bitwise approach similar to the procedure employed with Phase Encoding, eventually providing a bin-based binary-like representation of the input signal values. Such additional step, although increasing the total number of spikes, can result in a more robust encoding typical of spike bursts (Lisman, 1997).

##### 2.3.2.4. Latency/ISI

Neural communication through bursts of spikes, namely the increase from 1 to N of the number of spikes sent to carry information of a specific event, is known to improve reliability. However, also the latency between these N spikes, typically referred to as the inter-spike interval (ISI), can be taken into account to effectively encode information (Izhikevich et al., 2003). As a result, the Latency/ISI class of encoding algorithms is defined, with Burst Encoding as representative example.

###### 2.3.2.4.1. Burst encoding

As clearly pointed out in Guo et al. (2021), Burst Encoding is a well suited technique to carry information taking advantage of two different time-based characteristics of a single spike train. Such algorithm relies indeed on both the number of spikes and the ISI by employing the following three quantities: *N*_*max*_, namely the maximum number of spikes in each burst, *t*_*min*_, representing the minimum temporal distance between the spikes, and *t*_*max*_, which defines the maximum ISI value. By means of them, and by introducing the additional parameter *rate*, defined from a normalization procedure for each signal channel, the number of spikes and their relative distance are defined as:


(15) $$\begin{array}{l} SpikeNumber=\left\lceil rate\cdot N_{max}\right\rceil \end{array}$$

(16) $$ISI=\{\begin{array}{ll} \left\lceil t_{max}-rate(t_{max}-t_{min})\right\rceil & \text{ if }SpikeNumber>1\\ t_{max} & \text{ otherwise} \end{array}$$

Similarly to the previous two algorithm classes, Burst Encoding also results in spike trains with single polarity.

### 2.4. Transfer learning

To date, there are numerous algorithms for SNN training, which can be based on a global or local method. Global learning approaches consist in updating all the hyperparameters of the network at each training step, similarly to the classical approach applied for ANN architectures; such algorithms include Spike-Time-Dependent Plasticity (Kheradpisheh et al., 2018) and Back-Propagation Through Time (Lee et al., 2016). By contrast, in local approaches, only a subset of the hyperparameters are modified at each step; examples of these are Hebbian learning (Hebb, 1950) and E-prop (Bellec et al., 2020).

In the field of neuromorphic state-of-the-art for classification of audio signals (Acharya et al., 2018; Anumula et al., 2018; Dominguez-Morales et al., 2018; Ceolini et al., 2019), the method most often used to train an SNN is the Transfer learning approach. This technique is performed by training an artificial neural network (ANN) and subsequently porting the resulting weights to a spiking network of identical structure (Turner et al., 2022). The steps here applied to perform the training of the network and subsequently classify the samples are shown in Figure 1. Starting from the raw data, the time-varying signal is decomposed into different frequency channels through a bank of filters, whose structure and type mimics the ability of the cochlear hair cells in the human ear to decompose audio signals. The structure of the filter bank is as described in Ambikairajah et al. (2001): a battery of parallel band-pass filters, either of the Butterworth (Kayser et al., 2009) or gammatone (Ambikairajah et al., 2001; Katsiamis et al., 2006; Dennler et al., 2021) type. Each individual frequency channels is then encoded, using one of the methods introduced in Section 2.3, obtaining a translation of the original signal into the spike domain. In order to proceed with the training and classification process, a feature extraction phase is applied producing the sonogram, a reprocessing of the encoded spike-domain signal in the form of an image. For the creation of the sonogram, we employ the procedure described in Anumula et al. (2018), using the *Time Binning* process, which converts spike signals into frame-based features by counting the events over non-overlapping, fixed-length time windows.

In order to validate the transfer of the parameters obtained during CNN training into the SNN, it is necessary to use some precautions in the selection of the layer structure and the perceptor model, so as to guarantee equivalent behavior in the two architectures. Employing the method suggested by Liu et al. (2017) and applied in Dominguez-Morales et al. (2018) for the CNN architecture, a pooling layer implements an average pooling operation. The neuron model used in the convolutional and fully connected layers is the modified ReLU described in Liu et al. (2017), and in the output layer we use the Softmax activation function. Finally, the bias parameter is set equal to 0 for all neurons. At this point, as shown in the bottom left of Figure 1, the sonograms are presented as input data to the CNN. The network's training can then be performed through any of the classic methods, such as the Back-propagation or Stochastic gradient descent algorithm. Once the CNN training process is completed, a “twin” SNN network is built; this new network employs the Leaky Integrate and Fire (LIF) neuron model, while the synaptic weights are set equal to those extracted from the CNN. A final Poisson Rate encoding step is applied to the sonogram, in order to adapt the data to the input layer of the SNN. This procedure allows to obtain a uniform and fair evaluation method for the various coding techniques, since all encoded data are processed through the same procedure.

### 2.5. Model compression

As reported in Figure 1, model compression is the last step of the process: at this point, various optimization techniques may be employed to reduce the size of the network. In this work, we applied two successive phases: *synapse reduction*, which allows to selectively reduce the number of connections between neurons, and *fine-tuning*, where the remaining parameters of the network are optimized. The advantages in applying model compression are many, from a qualitative and quantitative point of view: obtaining a much smaller network, suitable for embedded systems thanks to a reduced memory footprint and a smaller computational cost, making the simulation of the network in non-neuromorphic hardware faster, and, in some cases, improving the accuracy due to the reduction of the overall stimulus transmitted through the synapses, which can introduce noise during the classification process.

The synapse reduction process carried out here consists in a selective elimination of the connections between neurons in all layers except the pooling layer, based on the weight associated to the synapse. Without changing the network's structure from the perspective of the number of layers and the neurons contained in each, this elimination is applied by calculating the distribution of the values for synaptic weights and then eliminating synapses with gradually greater weight. This procedure allows to eliminate synapses which contribute in a marginal way to the production of spikes, since, in the case of excitatory connections, a greater weight associated with a synapse corresponds to greater excitability of the neuron when stimulated. The reverse behavior occurs in inhibitory synapses.

After the synapse reduction process, the classification accuracy of the network generally worsens. In order to restore the original classification performance or even improve on it, a fine-tuning step is applied: constraining to 0 the eliminated weights, a CNN with the remaining connections is retrained for 5 epochs, and finally the weights are transferred back to the SNN version.

## 3. Results

In this section, we present the results of experiments aimed at evaluating several metrics of the signal processing, encoding and classification techniques presented in Section 2. We tested different configurations of the pipeline described in Figure 1 in an attempt to characterize the various encoding techniques' ability to handle time-varying input data. The resulting comparisons are proposed here not to highlight a single catch-all solution, but in order to provide detailed information to developers wanting to select the most suitable methods for their desired application; to that end, we benchmarked the pipeline configurations on two very different types of input data: the FSD and WISDM datasets.

The parameters we considered in order to tune the pipeline can be divided into two broad categories: in Section 3.1, we analyze *input encoding and processing* methods, including all elements contributing to the conversion of the data into the spike domain, and in Section 3.2 we characterize the impact of *architectural parameters*, focusing on the optimization of the network structure and its reduction through model compression.

### 3.1. Input encoding and processing

#### 3.1.1. Frequency decomposition

**Figure 4:**
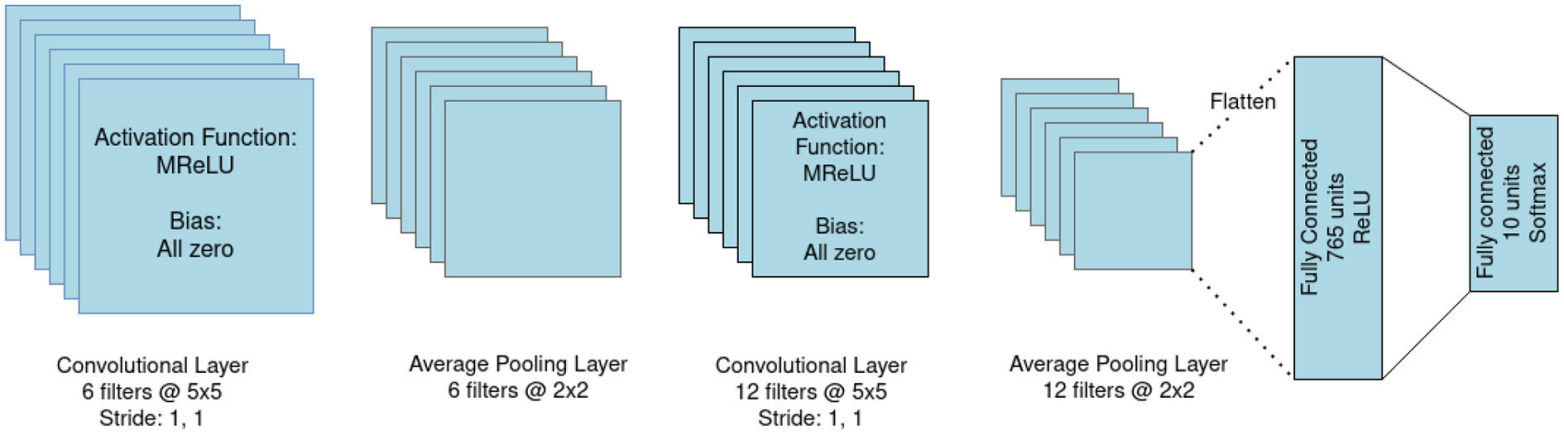
Architecture of the C6-C12-F2 convolutional neural network.

Decomposing the input signal into frequency channels can influence the encoding performance, increasing the amount of extractable features and producing a sonogram with a richer amount of information. We ran comprehensive tests comparing the impact of input frequency filtering on the accuracy of an sCNN performing classification of the FSD and WISDM datasets. The network used consists of 1 convolutional layer with *I* feature maps, 1 average pooling, 1 convolutional layer with *J* feature maps, 1 average pooling, and *K* fully-connected layers; we identify each variation on this structure with the acronym C*I*-C*J*-F*K*. A sample architecture, portraying configuration C6-C12-F2, is portrayed in Figure 4.

In our experiments, the gammatone filter demonstrates a better performance than the Butterworth filter. When classifying input data from the FSD, we test decomposition with 32 and 64 channels, recording throughout all channel configurations for the C6-C12-F2 architecture a median accuracy of 77.50 % for the Butterworth filter and 84.00 % for the gammatone. We observe particularly good classification accuracy with Phase Encoding, reporting 83.00 % with Butterworth filters and reaching 93.00 % in the case of gammatone. The latter result is due to redundant components present in the frequency response that lead to a higher number of spikes for this algorithm class, allowing to encode more information. For the WISDM dataset, due to its reduced sampling frequency, only the 4-, 8-, and 16-channel separation configurations could be tested. For all encoding types, lower average test accuracy rates are observed than with the FSD: 66.67 % with the Butterworth filter and 46.67 % for the gammatone, both coupled with a C12-C24-F2 network architecture.

#### 3.1.2. Comparing different classes of encoding algorithms

**Figure 5:**
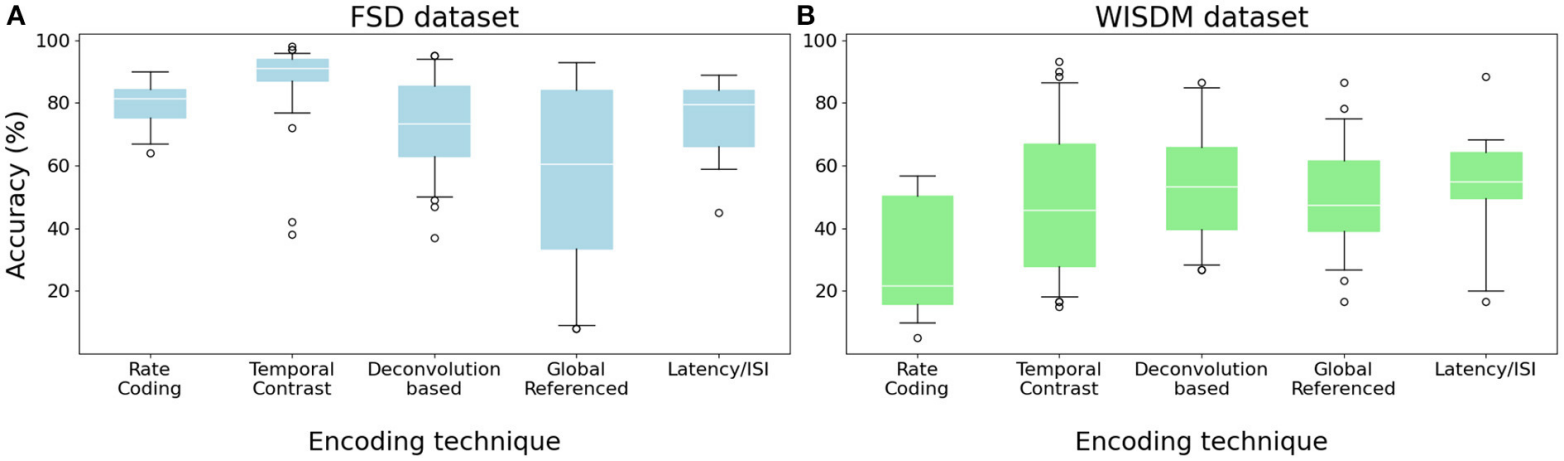
Median accuracy values for each encoding class, given different combinations of network architecture, filter type, number of channels and feature extraction bins. **(A)** FSD dataset. **(B)** WISDM dataset.

While the encoding step is fundamental and necessary in order to use digital input data with an SNN, choosing the most suitable encoding technique for the signal to be analyzed can also improve accuracy. Figure 5 shows a comparison of the median accuracy reached by different families of encoding algorithms combined with all different channel separations, feature extraction methods and network architectures. For the FSD, the Temporal Contrast class presented the best accuracy, having a median of about 91.00 % (Figure 5A). On the other hand, the Global Referenced class reports the worst median result—around 53 %—with a high variance. This is due to the very different performance of the two algorithms in the Global Referenced family: while Phase Encoding yields acceptable results (median 77.5 %, with a maximum of 93 %), TTFS reports very low accuracy (median 35 %, with a minimum of 8 %). This is likely due to the reduced number of spikes produced by TTFS, leading to insufficient stimulation of the network: we will explore this concept in greater detail in the next subsection.

When performing classification of the WISDM dataset, while the different algorithms obtain quite heterogeneous results, the median accuracy aggregated by algorithm class remains around 48 % for all classes except Rate Coding, which obtains the worst median results at 21.67 % and the overall minimum at 5 %. The best median result is achieved by Burst Encoding with 55 % accuracy, while the single best result is obtained, at 93 %, by the ZCSF algorithm combined with a 16-channel Butterworth filter and a C6-C12-F2 network architecture.

##### 3.1.2.1. Spike density

**Figure 6:**
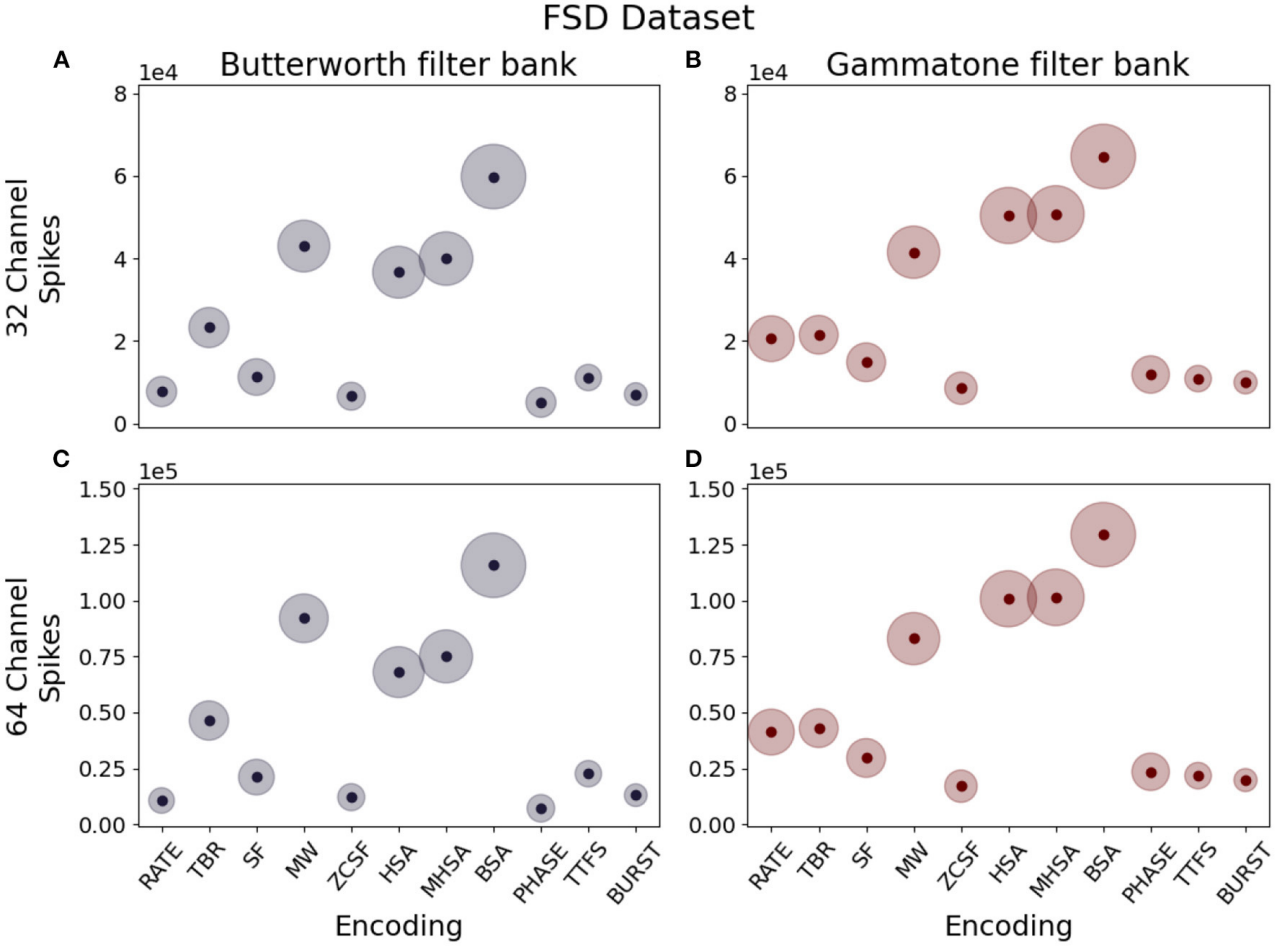
Median spike counts per sample generated by different combinations of encoding techniques, number of channels and filter types for the FSD dataset. **(A)** Butterworth filter, 32 channels. **(B)** Gammatone filter, 32 channels. **(C)** Butterworth filter, 64 channels. **(D)** Gammatone filter, 64 channels.

**Figure 7:**
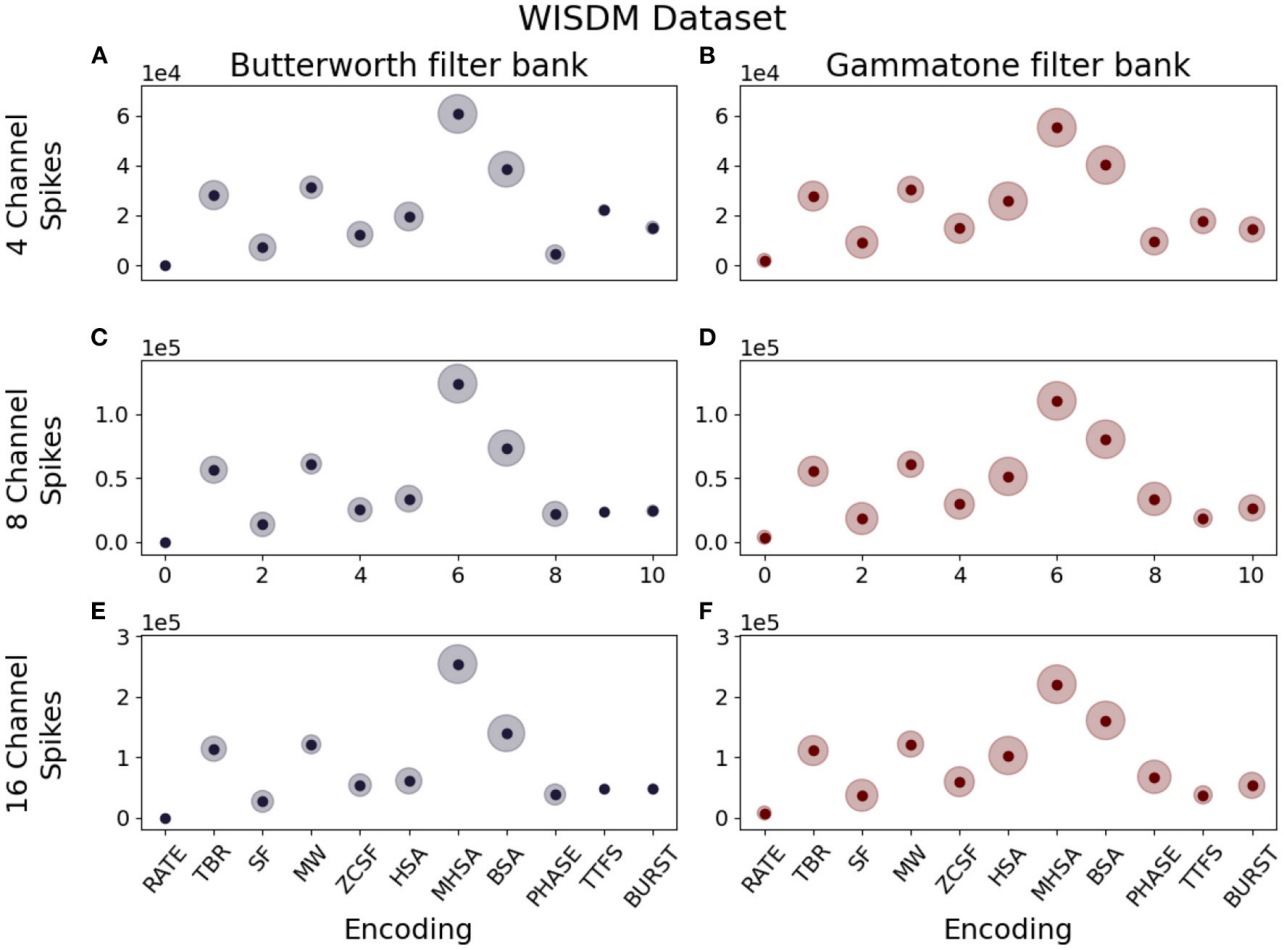
Median spike counts per sample generated by different combinations of encoding techniques, number of channels and filter types for the WISDM dataset. **(A)** Butterworth filter, 4 channels. **(B)** Gammatone filter, 4 channels. **(C)** Butterworth filter, 8 channels. **(D)** Gammatone filter, 8 channels. **(E)** Butterworth filter, 16 channels. **(F)** Gammatone filter, 16 channels.

Spike density is defined as the amount of spikes produced per unit time. This parameter should be carefully considered when designing a neuromorphic system, since a lower spike density leads to energy savings thanks to reduced communication between layers of the network, but a too low number of spikes can prove insufficient to efficiently encode information without loss. Our observations show that this quantity is greatly influenced by the encoding algorithm: given the same input data, the different implementation logic of each algorithm results in different spike densities. The box plots in Figures 6, 7 describe the distribution of spikes generated by each coding technique after channel separation by a Butterworth (left column) or gammatone (right column) filter bank. In all considered cases, the Deconvolution-based family of encoding algorithms (HSA, MHSA, BSA) produces the highest spike count.

Including the refractory period in the encoding model also influences the amount of spikes produced. We performed preliminary experiments using different values for the refractory period τ_*ref*_: 3 ms, 2 ms and 1 ms. In all cases, the use of this parameter in the encoding step leads to an excessive reduction of the number of spikes, such that the layers of the SNN could not be sufficiently stimulated. This causes a drastic reduction of the classification performance: the median test accuracy for the FSD for all architectures, channel decomposition and encoding techniques is 22.00 %. In the case of WISDM, the value of τ_*ref*_ is bounded by the low sampling frequency *f*_*s*_= 20 Hz of the dataset signals, leading to a lower bound of 50 ms. Due to the performance deterioration we found even with small values for τ_*ref*_, all results reported within this document are obtained using τ_*ref*_ = 0.

#### 3.1.3. Feature extraction

**Figure 8:**
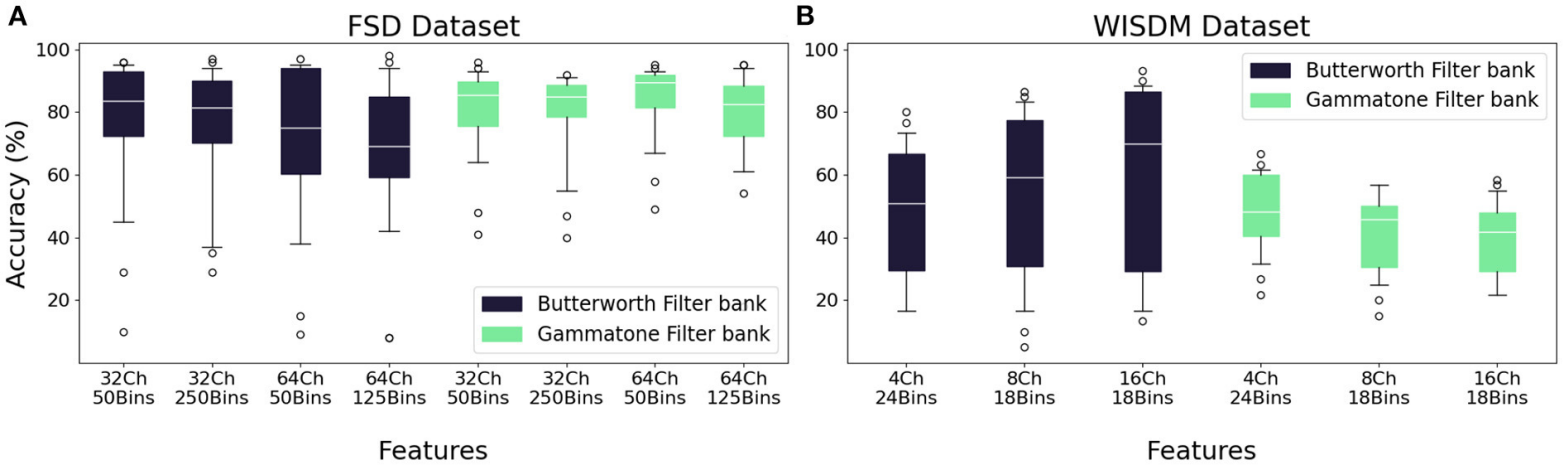
Median accuracy values for each feature extraction class, given different combinations of network architecture and encoding techniques. **(A)** FSD dataset. **(B)** WISDM dataset.

**Figure 9:**
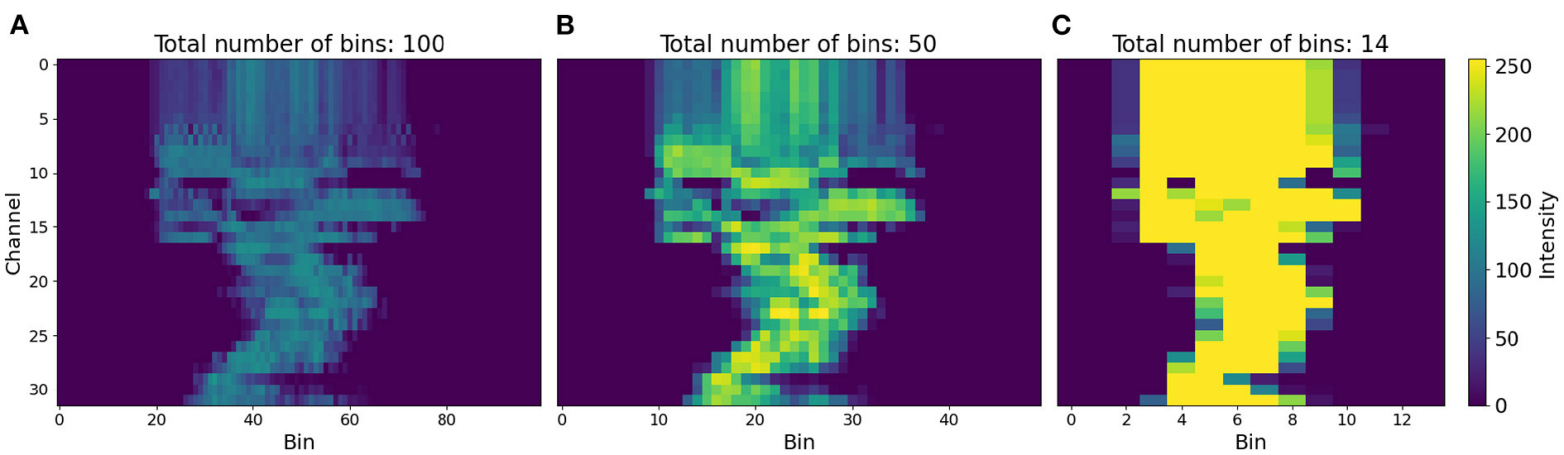
Visual representation of a 32-channel sonogram processed into 100, 50, and 14 time bins. The 50-bin subdivision strikes the best balance of resolution and information density. **(A)** Total number of bind: 100. **(B)** Total number of bind: 50. **(C)** Total number of bind: 14.

The feature extraction step is necessary in order to use the transfer learning method with a non-spiking CNN model, and consists of the production of the sonogram, a binned representation of the input suitable for elaboration by convolutional layers. We borrow this term from Dominguez-Morales et al. (2018); while it was originally used to describe the binned representation of an audio signal, hence the word, we apply the same definition for the corresponding representation of the WISDM dataset as well as for the FSD. The number of bins, i.e., the number of intervals in which the spike-coded signal is to be divided, is the parameter that determines the resolution of the sonogram and the quality of the feature extraction. We tested several values in order to identify the best separation into bins. In the case of the FSD dataset, the tested binning intervals are 50 and 250 for the 32-channel filter bank, 50 and 125 for the 64-channel filter bank. For the WISDM dataset, we have, respectively, 24, 18, 18 bins for the separation into 4, 8, and 16 channels; we selected only one binning type for each channel separation, because other values showed unsatisfactory accuracy performances. The results of the comparison are reported in Figure 8. As previously seen in Section 3.1.1, overall worse accuracies are observed for the WISDM dataset. Finally, regardless of the number of channels selected for the pre-processing step, overall worse performances are obtained for high bin counts. In fact, too large or too small values for this parameter result in a quasi-uniform pattern with reduced information, as the difference in intensity between the pixels of the sonogram becomes too small; an example is shown in Figure 9.

### 3.2. Architectural parameters

#### 3.2.1. CNN/SNN architecture

The type and structure of the classification network is another element that can affect accuracy performance. The network architecture we selected to perform initial training and enable transfer learning is the convolutional neural network (CNN). This choice was made based on the state-of-the-art results previously reached by this class of network in the classification of audio signals recorded by a neuromorphic cochlea (Dominguez-Morales et al., 2018). Further experiments conducted by the authors (Fra et al., 2022) confirmed the CNN's computational and energetic efficiency in the analysis of time-varying signals.

We developed several test configurations for the CNN structure, starting from the work presented in Dominguez-Morales et al. (2018) and performing structural hyperparameter optimization. The tested networks all present the basic structure presented in Figure 4, while varying the number of filters in the 2 convolutional layers and the number of fully-connected layers. All networks were trained by transfer learning and the intermediate results of the corresponding ANNs are reported in the Supplementary material.

The top performing networks are C12-C24-F1, C6-C12-F2, and C12-C24-F2. For classification of the FSD, the median accuracy reported by the C12-C24-F1 configuration is 53.00 %, while the other two networks perform substantially better, obtaining 82.50 % for C6-C12-F2 and 84.00 % for C12-C24-F2. C6-C12-F2 and C12-C24-F2 are also the best-performing architectures for the WISDM dataset, obtaining median accuracies of 45.00 and 52.50 % respectively. The reason for these low values is not intrinsically tied to the structure of the network, but, as seen in previous sections, it is most likely due to a lesser efficiency of the examined encoding algorithms with this kind of low-frequency data.

#### 3.2.2. Model compression

**Figure 10:**
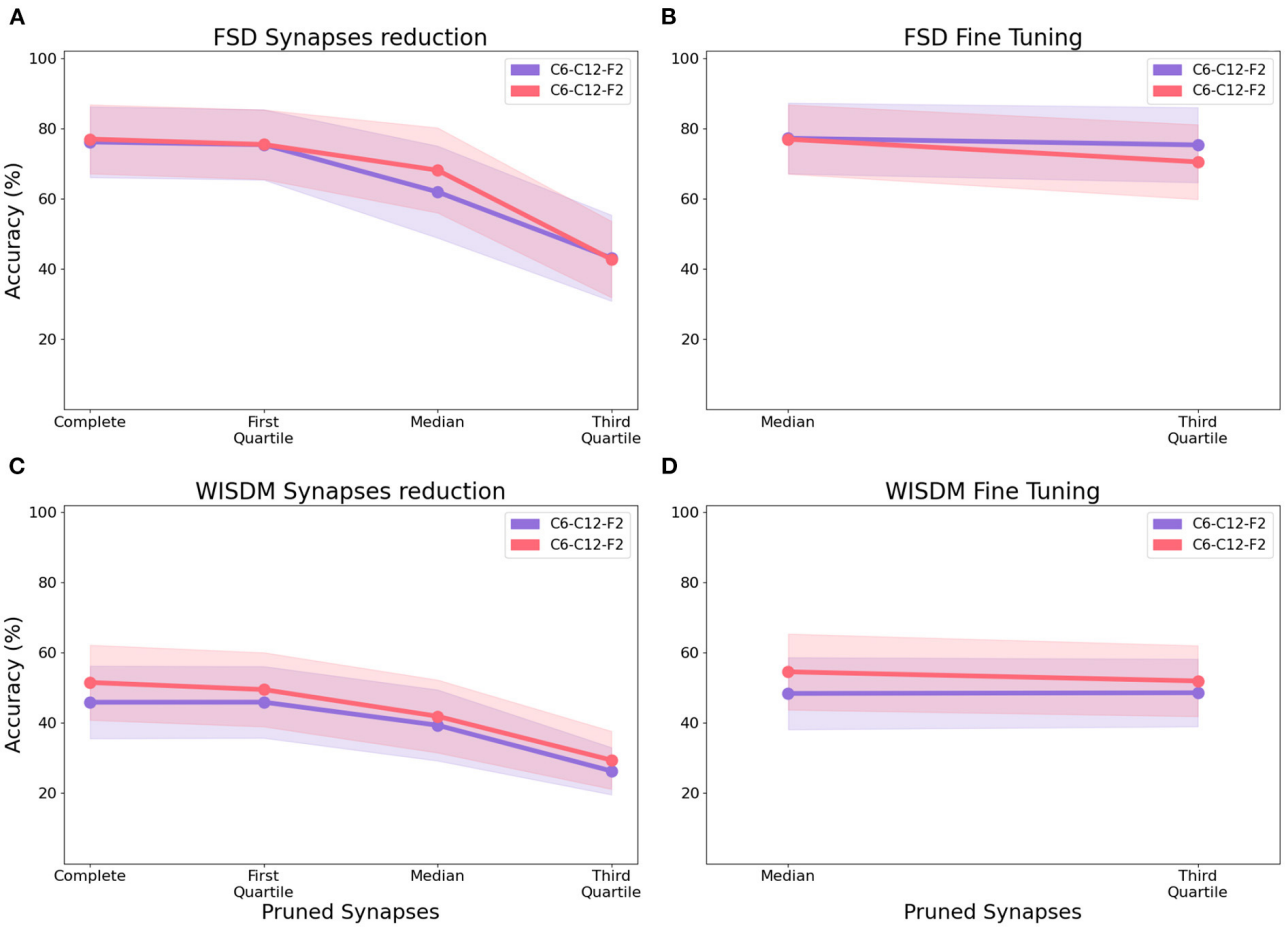
Median test accuracy of all encoding class, filter type, number of channels, feature extraction bins configurations for architectures C6-C12-F2 and C12-C24-F2 performing classification of the FSD and WISDM datasets, after synapse reduction **(A,C)** and after fine tuning **(B,D)**.

After identifying the best-performing CNN structure, we apply model compression techniques aimed at reducing the connectivity within the network, thereby reducing its memory, computation and energy requirements. In order to test the effectiveness of the aforementioned techniques, we apply the synapse reduction process to the best-performing network configurations from previous experiments: C6-C12-F2 and C12-C24-F2. We progressively eliminate connections with increasing synapse weights based on the distribution of their absolute values: first we remove connections whose weight is less than or equal to the first quartile, then to the median, and finally to the third quartile. Figure 10A shows the impact of synapse reduction on the classification of the FSD dataset: the more connections removed, the worse the classification performance. This trend is due to the reduction in the number of spikes in the network, making it difficult to correctly stimulate the neurons in the fully connected layers. In order to optimize the model described by the residual synapses, a fine-tuning process is applied to the smallest network (C6-C12-F2) by copying the connection settings back to the original CNN and retraining it for around 10–20 epochs. Once the retrained weights are transferred to the final version of the sCNN, an accuracy comparable to the complete network is recorded, with a few configurations slightly outperforming the original network by as much as 1.75 %. The compressed networks reach a median test accuracy of 81.00 % while retaining only 25 % of the original network size by pruning up to the third quartile (Figure 10B); this median value is obtained across all filter bank, feature extraction and encoding algorithm configurations for the given architecture.

**Figure 11:**
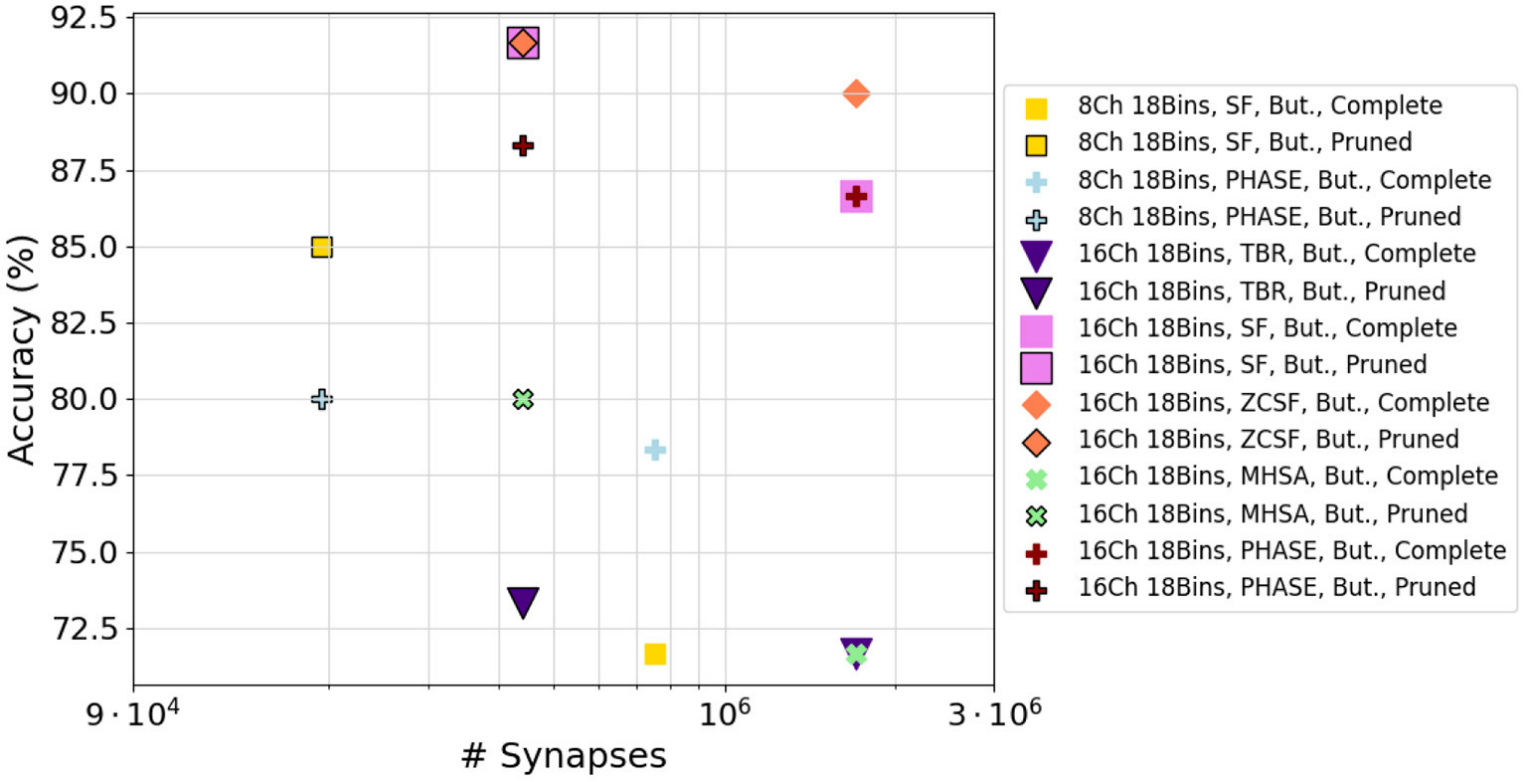
A summary of network configurations that achieved improved performance after model compression, in the case of WISDM dataset.

In the case of WISDM, synapse reduction also causes a reduction of the accuracy (Figure 10C). However, after applying the fine-tuning of the network to the C12-C24-F2 architecture (Figure 10D), an increase in the maximum achievable accuracy can be recorded for certain configurations, resulting in better performance for the reduced network than for the complete one. For example, the ZCSF algorithm for 16 channels, 18 bins with 3rd-quartile synapse reduction obtains an increment of 1.7 to 91.7 %; the SF algorithm for 16 channels, 18 bins with median synapse reduction achieves an increment of 8.3 %, reaching an accuracy of 95.0 %. This improvement can be traced back to the combined effect of synapse reduction and fine-tuning, allowing to reduce the number of connections in the network while maintaining a model suitable for the representation of the data: this causes a reduction in the noise traveling through the network, with beneficial effects on the classification process. Configurations that improve their performance after model compression are portrayed in Figure 11.

## 4. Discussion

We performed a detailed benchmarking of different possible combinations of frequency decomposition filters, encoding algorithm, feature extraction parameters and network architectures, coupled with transfer learning and a spiking CNN. The aim of the work is to compose a sort of *vademecum* providing neuromorphic engineers with valuable information on the comparative performance of various encoding techniques. Applying the same pipeline to two different datasets, our experiment highlighted the importance of tailoring the encoding type to the input data. Indeed, the performance of the considered encoding techniques depends on the frequency of the input data. For the middle-frequency FSD dataset, having a wider bandwidth, more features can be extracted form the signal, and it is easier to spot the encoding classes that enable more accurate classification. On the other hand, for very-low frequency data like the WISDM dataset, there is no clear advantage for a given algorithm class over the others; however, several configurations featuring Temporal Coding such as ZCSF encoding vastly outperformed Rate-based Coding, demonstrating that while the algorithm for the encoding must be carefully chosen, Temporal Coding has a higher ability to extract from a very-low frequency signal features suitable for analysis in the neuromorphic domain. We also observed that the spike count produced by each coding must be sufficiently high to stimulate all layers of the downstream SNN properly, therefore the reduction of spike count aimed at power savings must be carefully balanced with the retention of information.

**Figure 12:**
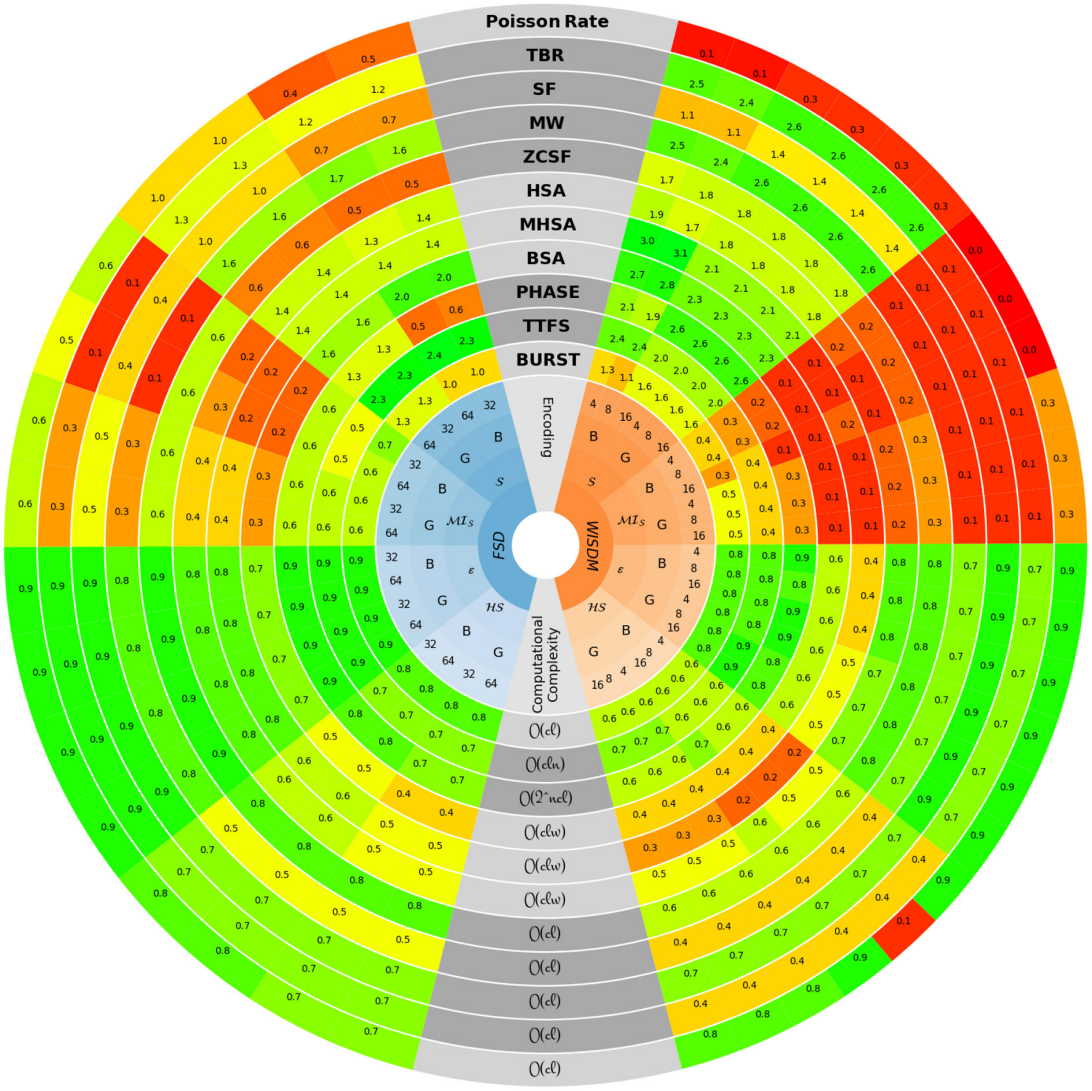
The characterization of each encoding technique is presented along specific rings of the circle-shaped graph. The bottom, central part reports the computational complexity, defined through the quantities *l* (signal length), *c* (number of channels), *n* (length of the bitwise representation) and *w* (width of the convolution function). The left-hand side refers to the values achieved on FSD data, while the right-hand side refers to those obtained with the WISDM dataset. The four signals-related metrics, namely S, $MI_{S}$, HS and ε, are shown in a mirrored arrangement with respect to the vertical symmetry axis of the circle. For each of them, the results provided by the two filter types, i.e., Butterworth (B) and Gammatone (G), are reported according to the number of channels used to split the original signal.

In Figure 12, a quantitative and comparative overview of all the investigated encoding techniques is presented. Each of them is characterized through five different metrics: Shannon entropy S of the encoded signal (Shannon, 1948), mutual information of the encoded signal with the original input (Quian Quiroga and Panzeri, 2009) normalized with respect to entropy $MI_{S}$, sparsity HS of the encoded signal (Hoyer, 2004), spiking efficiency ε (Dupeyroux et al., 2021) and computational complexity O(*f*). All the results are summarized in Supplementary Table S1.

In Table 1, we report a summary of recommendations matching each encoding technique to the input frequency of the time-variant input data. We present this table as the focal result of this work, constituting a first step toward extensive comparison of the tools available for signal representation in the neuromorphic domain. As commercial interest for this area of study increases, we foresee a growing necessity for this type of research providing guidelines for the solution of engineering problems in the realm of IoT and Industry 4.0.

**Table 1:** Summary of encoding techniques, taking into account their performances with respect to the type of input data.

**Encoding class and technique**			**Temporal data**		**Spatial data^a^**

			**Very-Low frequency**	**Middle frequency**	
Rate coding	Poisson rate		✗	✓	✓
Temporal coding	Temporal contrast	TBR	✓	✓	✗
		SF	✓	✓	✗
		MW	✓	✓	✗
		ZCSF	✓	✓	✗
	Filter and optimizer	HSA	−	−	✗
		MHSA	−	−	✗
		BSA	✓	−	✗
	Global referenced	PHASE	✗	✓	✓
		TTFS	✗	✓	✓
	Latency/ISI	BURST	✗	✓	✓

A ✓indicates the technique is particularly suitable for the purpose, while −means the technique presents some disadvantages and ✗that it is not suitable for the purpose.^a^Guo et al. (2021) and Auge et al. (2021).

In future work, we plan to expand this study by including in the comparison novel encoding methods directly performed by neuronal input layers embedded within a SNN. We will also consider spiking input data produced by event-based sensors such as silicon cochleas. As more and more options appear in the field of neuromorphic encoding, benchmarking studies (Stewart et al., 2015; Blouw et al., 2019; Davies, 2019; Forno et al., 2021a) are on their way to becoming a valuable tool in guiding research and development toward the most suitable solution for any given application.

## Data availability statement

Publicly available datasets were analyzed in this study. This data can be found at: WISDM (https://archive.ics.uci.edu/ml/datasets/WISDM+Smartphone+and+Smartwatch+Activity+and+Biometrics+Dataset+) and FSD (https://github.com/Jakobovski/free-spoken-digit-dataset).

## Author contributions

EF, VF, RP, and GU designed the analysis and wrote the manuscript. RP implemented the test pipeline, ran experiments, and gathered results. GU and EM supervised the research work. All authors participated in reviewing the manuscript and approved the submitted version.

## Footnotes

^1^ https://docs.edgeimpulse.com/docs/what-is-embedded-machine-learning-anyway

^2^ https://www.st.com/en/partner-products-and-services/qeexo-automl-embedded-machine-learning-tool.html

^3^ https://microsoft.github.io/ELL/

^4^ https://github.com/Jakobovski/free-spoken-digit-dataset

^5^ https://www.sony.com/en/SonyInfo/sony_ai/technology/evs.html
