# Supplementary material for: Spike encoding techniques for IoT time-varying signals benchmarked on a neuromorphic classification task
Source: Front Neurosci. 2022 Dec 21;16:999029. doi: 10.3389/fnins.2022.999029 (PMC9811205; doi:10.3389/fnins.2022.999029)
Supplement: Supplementary file 1 [file Data_Sheet_1.PDF]

Supplementary Material

1 BUTTERWORTH FILTERS

Every class of filters is identified by a characteristic transfer function, which defines properties and technical details of the filtering activity. When the latter can be described by means of a Butterworth polynomial $B^{(n)}(s/\omega_0)$, where n is the degree, the corresponding filter belongs to the class of Butterworth filters, and its transfer function can be written as:

$$H(s) = \frac{B^{(n)}(\frac{s}{\omega_0})}{B^{(m)}(\frac{s}{\omega_0})} \tag{S1}$$

with $m > n$ and the Butterworth polynomial defined as:

$$B_n(s) = \sum_{k=0}^{n} a_k s^k \tag{S2}$$

where

$$a_k = \prod_{l=1}^{k} \frac{\cos((l-1)\gamma)}{\sin(l\gamma)} \tag{S3}$$

with $a_0 = 1$ and $\gamma = \frac{\pi}{2n}$.

2 GAMMATONE FILTERS

Explicitly inspired to the Gamma-tone introduced by Aertsen and Johannesma in (Aertsen and Johannesma, 1980)

$$f(t) = A \cdot m(t) \cdot \cos(\omega t + \varphi) \tag{S4}$$

where A is the amplitude factor, ω is the angular carrier frequency, φ is the starting phase and $m(t)$ is defined as

$$\begin{aligned} m(t) &= c\left(\frac{t}{\beta}\right)^{\gamma-1} e^{\frac{-t}{\beta}} \qquad \text{for } t \geq 0 \text{ and } \beta > 0 \\ m(t) &= 0 \qquad \text{for } t < 0 \text{ and } \gamma \geq 0 \end{aligned} \tag{S5}$$

with β being the duration parameter, γ the form parameter and c the normalization constant giving $m^2(t)$ unit area; gammatone filters are described by the following transfer function:

$$H(s) = \frac{e^{j\varphi}\left[s + \frac{\omega_0}{2Q} + j\omega_0\sqrt{1 - \frac{1}{4Q^2}}\right]^N + e^{-j\varphi}\left[s + \frac{\omega_0}{2Q} - j\omega_0\sqrt{1 - \frac{1}{4Q^2}}\right]^N}{[s^2 + \frac{\omega_0}{Q}s + \omega_0^2]^N} \tag{S6}$$

where ω_0 is the natural, or pole, frequency, Q is the quality factor and N is the order.

3 PSEUDOCODE OF THE ADOPTED ENCODING TECHNIQUES

Algorithm 1 Poisson Rate

```
input:   Signal (n × m matrix), Duration (double), Silence (double)
output:  SpikeTrain

Rows ← n
Cols ← m
SpikeTrain ← EmptyMatrix
for r = 1 : Rows do
    for c = 1 : Cols do
        rate ← Signal[r][c]
        if rate == 0 then
            SpikeTrain ← append(EmptyArray)
        else
            SpikeSequence ← EmptyArray
            ISI ← −log(1.0 − random())/rate ∗ 1000.0
            SpikeTime ← ISI
            while SpikeTime < Duration do
                SpikeSequence ← append(SpikeTime)
                ISI ← −log(1.0 − random())/rate ∗ 1000.0
                SpikeTime ← SpikeTime + ISI
            end while
            SpikeTrain ← append(SpikeSequence)
        end if
    end for
end for
```

Algorithm 2 Threshold-Based Representation (TBR)

input: $Signal$ ($n \times m$ matrix), γ (double), τ_{ref} (double), Δt (double)
output: $SpikeTrain$

$Channels \leftarrow n$
$L \leftarrow m$
$SpikeTrain \leftarrow zeros(Channels, L)$
$SpikeTime \leftarrow linspace(0, \Delta t, L)$
$t_{spike} \leftarrow 0$
for $c = 1 : Channels$ **do**
  $Variation \leftarrow zeros(1, L)$
  for $i = 1 : L - 1$ **do**
    $Variation[i] \leftarrow Signal[c][i+1] - Signal[c][i]$
  end for
  $Threshold = mean(Variation) + \gamma \cdot std(Variation)$
  $Variation \leftarrow [Variation[1], Variation]$
  for $i = 1 : L$ **do**
    if $i == 1$ **then**
      if $Variation[i] > Threshold$ **then**
        $SpikeTrain[c][i] \leftarrow 1$
      else if $Variation[i] < -Threshold$ **then**
        $SpikeTrain[c][i] \leftarrow -1$
      end if
    else if $SpikeTime[i] - t_{spike} > \tau_{ref}$ **then**
      if $Variation[i] > Threshold$ **then**
        $SpikeTrain[c][i] \leftarrow 1$
        $t_{spike} \leftarrow SpikeTime[i]$
      else if $Variation[i] < -Threshold$ **then**
        $SpikeTrain[c][i] \leftarrow -1$
        $t_{spike} \leftarrow SpikeTime[i]$
      end if
    end if
  end for
end for

Algorithm 3 Moving Window (MW)

input: $Signal$ ($n \times m$ matrix), $Window$ (int), τ_{ref} (double), Δt (double)
output: $SpikeTrain$

```
Channels ← n
L ← m
SpikeTrain ← zeros(Channels, L)
SpikeTime ← linspace(0, Δt, L)
t_spike ← 0
for c = 1 : Channels do
    Variation ← zeros(1, L)
    for i = 1 : L − 1 do
        Variation[i] ← abs(Signal[c][i + 1] − Signal[c][i])
    end for
    Threshold = mean(Variation)
    Base = mean(Signal[c][1 : Window])
    for i = 1 : L do
        if i ≤ Window then
            if Signal[c][i] > Base + Threshold then
                SpikeTrain[c][i] ← 1
            else if Signal[c][i] < Base − Threshold then
                SpikeTrain[c][i] ← −1
            end if
        else if SpikeTime[i] − t_spike > τ_ref then
            Base = mean(Signal[c][i − Window : i])
            if Signal[c][i] > Base + Threshold then
                SpikeTrain[c][i] ← 1
                t_spike ← SpikeTime[i]
            else if Signal[c][i] < Base − Threshold then
                SpikeTrain[c][i] ← −1
                t_spike ← SpikeTime[i]
            end if
        end if
    end for
end for
```

Algorithm 4 Step-Forward (SF)

input: $Signal$ ($n \times m$ matrix), γ (double), τ_{ref} (double), Δt (double)
output: $SpikeTrain$

```
Channels ← n
L ← m
SpikeTrain ← zeros(Channels, L)
SpikeTime ← linspace(0, Δt, L)
t_spike ← 0
Jump ← zeros(Channels)
for c = 1 : Channels do
    Jump[c] ← max(Signal[c]) − min(Signal[c])
end for
Threshold ← mean(Jump)/γ
for c = 1 : Channels do
    Base ← Signal[c][1]
    for i = 1 : L do
        if SpikeTime[i] − t_spike > τ_ref then
            if Signal[c][i] > Base + Threshold then
                SpikeTrain[c][i] ← 1
                Base = Base + Threshold
                t_spike ← SpikeTime[i]
            else if Sample[c][i] < Base − Threshold then
                SpikeTrain[c][i] ← −1
                Base = Base − Threshold
                t_spike ← SpikeTime[i]
            end if
        end if
    end for
end for
```

Algorithm 5 Zero-Crossing Step-Forward (ZCSF)

input: $Signal$ ($n \times m$ matrix), γ (double), τ_{ref} (double), Δt (double)
output: $SpikeTrain$

$Channels \leftarrow n$
$L \leftarrow m$
$SpikeTrain \leftarrow zeros(Channels, L)$
$SpikeTime \leftarrow linspace(0, \Delta t, L)$
$t_{spike} \leftarrow 0$
$Jump \leftarrow zeros(Channels)$
for $c = 1 : Channels$ **do**
    $Jump[c] \leftarrow \max(Signal[c]) - \min(Signal[c])$
end for
$Threshold \leftarrow mean(Jump)/\gamma$
for $c = 1 : Channels$ **do**
    for $i = 1 : L$ **do**
        if $SpikeTime[i] - t_{spike} > \tau_{ref}$ **then**
            if $Signal[c][i] > Threshold \;\&\; Signal[c][i] > 0$ **then**
                $SpikeTrain[c][i] \leftarrow 1$
                $t_{spike} \leftarrow SpikeTime[i]$
            end if
        end if
    end for
end for

Algorithm 6 Hough Spiker Algorithm (HSA)

input: $Signal$ ($n \times m$ matrix), $filter$ (k array), τ_{ref} (double), Δt (double)
output: $SpikeTrain$

```
Channels ← n
L ← m
L_filter ← k
SpikeTrain ← zeros(Channels, L)
SpikeTime ← linspace(0, Δt, L)
t_spike ← 0
for c = 1 : Channels do
    for i = 1 : L do
        counter ← 0
        for j = 1 : L_filter do
            if i + j − 1 < L  &  Signal[c][i + j − 1] ≥ filter[j] then
                counter ← counter + 1
            end if
            if counter == L_filter then
                for j = 1 : L_filter do
                    if i + j − 1 < L then
                        Signal[c][i + j − 1] ← Signal[c][i + j − 1] − filter[j]
                    end if
                end for
                if SpikeTime[i] − t_spike > τ_ref then
                    SpikeTrain[c][i] ← 1
                    t_spike ← SpikeTime[i]
                end if
            end if
        end for
    end for
end for
```

Algorithm 7 Modified Hough Spiker Algorithm (MHSA)

input: $Signal$ ($n \times m$ matrix), $filter$ (k array), $Threshold$ (double), τ_{ref} (double), Δt (double)
output: $SpikeTrain$

```
Channels ← n
L ← m
L_filter ← k
SpikeTrain ← zeros(Channels, L)
SpikeTime ← linspace(0, Δt, L)
t_spike ← 0
for c = 1 : Channels do
    for i = 1 : L do
        error ← 0
        for j = 1 : L_filter do
            if i + j − 1 < L  &  Signal[c][i + j − 1] < filter[j] then
                error ← error + filter[j] − Signal[c][i + j − 1]
            end if
        end for
        if error ≤ Threshold then
            for j = 1 : L_filter do
                if i + j − 1 < L then
                    Signal[c][i + j − 1] ← Signal[c][i + j − 1] − filter[j]
                end if
            end for
            if SpikeTime[i] − t_spike > τ_ref then
                SpikeTrain[c][i] ← 1
                t_spike ← SpikeTime[i]
            end if
        end if
    end for
end for
```

Algorithm 8 Ben's Spiker Algorithm (BSA)

input: $Signal$ ($n \times m$ matrix), $filter$ (k array), $Threshold$ (double), τ_{ref} (double), Δt (double)
output: $SpikeTrain$

```
Channels ← n
L ← m
L_filter ← k
SpikeTrain ← zeros(Channels, L)
SpikeTime ← linspace(0, Δt, L)
t_spike ← 0
for c = 1 : Channels do
    for i = 1 : L do
        error1 ← 0
        error2 ← 0
        for j = 1 : L_filter do
            if i + j − 1 < L then
                error1 = error1 + abs(Signal[c][i + j − 1] − filter[j])
                error2 = error2 + abs(Signal[c][i + j − 1])
            end if
        end for
        if error1 ≤ error2 · Threshold then
            for j = 1 : L_filter do
                if i + j − 1 < L then
                    Signal[c][i + j − 1] = Signal[c][i + j − 1] − filter[j]
                end if
            end for
            if SpikeTime[i] − t_spike > τ_ref then
                SpikeTrain[c][i] ← 1
                t_spike ← SpikeTime[i]
            end if
        end if
    end for
end for
```

Algorithm 9 Phase encoding (PHASE)

input: $Signal$ ($n \times m$ matrix), β (integer), τ_{ref} (double), Δt (double)
output: $SpikeTrain$

```
level = lambda(x) : pi/(2^(β+1)) · x
Channels ← n
L ← m
L_red ← int(m/β)
Signal_red ← zeros(Channels, L_red)
SpikeTrain ← zeros(Channels, L)
SpikeTime ← linspace(0, Δt, L_red)
t_spike ← 0
for c = 1 : Channels do
    for i = 1 : L do
        if Signal[c][i] < 0 then
            Signal[c][i] ← 0
        end if
    end for
end for
for c = 1 : Channels do
    for i = 1 : β : L_red do
        Signal_red[c][i] ← mean(Signal[c][i : i + β])
    end for
end for
Normalization ← max(Signal_red)
for c = 1 : Channels do
    for i = 1 : L_red do
        if SpikeTime[i] − t_spike > τ_ref then
            for j = 0 : 2^β − 1 do
                if level(j) ≤ arcsin(Signal_red[c][i]/Normalization) ≤ level(j + 1) then
                    SpikeTrain[c][(i − 1) · β + 1 : i · β] ← reverse(bin(j))
                    t_spike ← SpikeTime[i]
                    break
                end if
            end for
        end if
    end for
end for
```

Algorithm 10 Time-to-First-Spike (TTFS)

input: $Signal$ ($n \times m$ matrix), β (integer), τ_{ref} (double), Δt (double)
output: $SpikeTrain$

```
level = lambda(x) : 1/β · x
Channels ← n
L ← m
L_red ← int(m/β)
SpikeTrain ← zeros(Channels, L)
SpikeTime ← linspace(0, Δt, L_red)
t_spike ← 0
Signal_red ← zeros(Channels, L_red)
for c = 1 : Channels do
    for i = 1 : L do
        if Signal[c][i] < 0 then
            Signal[c][i] ← 0
        end if
    end for
end for
for c = 1 : Channels do
    for i = 1 : β : L_red do
        Signal_red[c][i] ← mean(Signal[c][i : i + β])
    end for
end for
Normalization ← max(Signal_red)
for c = 1 : Channels do
    for i = 1 : L_red do
        if SpikeTime[i] − t_spike > τ_ref then
            for j = 0 : β − 1 do
                if level(j) ≤ 0.1 · log(Normalization/Signal_red[c][i]) ≤ level(j + 1) then
                    SpikeTrain[c][(i − 1) · β + j] ← 1
                    t_spike ← SpikeTime[i]
                    break
                end if
            end for
        end if
    end for
end for
```

Algorithm 11 Burst Encoding (BURST)

input: $Signal$ ($n \times m$ matrix), $Burst$ (integer), N_{max} (integer), t_{max} (integer), t_{min} (integer), τ_{ref} (double), Δt (double)
output: $SpikeTrain$

```
Channels ← n
L ← m
L_redux ← m/Burst
SpikeTrain ← zeros(Channels, L)
SpikeTime ← linspace(0, Δt, L_red)
t_spike ← 0
Signal_red ← zeros(Channels, L_red)
for c = 1 : Channels do
    for i = 1 : L do
        if Signal[c][i] < 0 then
            Signal[c][i] ← 0
        end if
    end for
end for
for c = 1 : Channels do
    for i = 1 : Burst : L do
        Signal_red[c][i] ← mean(Signal[c][i : i + Burst])
    end for
end for
Normalization ← max(Signal_red)
for c = 1 : Channels do
    for i = 1 : L_red do
        if SpikeTime[i] − t_spike > τ_ref then
            Rate ← Signal_red[c][i]/Normalization
            SpikeNumber ← ⌈Rate · N_max⌉
            if SpikeNumber > 1 then
                ISI ← ⌈t_M − Rate · (t_M − t_m)⌉
            else
                ISI ← t_M
            end if
            SpikeTrain[c][(i − 1) · Burst + 1 : i · Burst] ← ([1] + ISI · [0]) · SpikeNumber
            t_spike ← SpikeTime[i]
        end if
    end for
end for
```

4 ENCODING ALGORITHMS CHARACTERIZATION

The investigated encoding algorithms have been compared through five different metrics: computational complexity $\mathcal{O}(f)$ and four quantities accounting for signals properties and information content, namely Shannon entropy $\mathcal{S}$ of the encoded signal (Shannon, 1948), mutual information of the encoded signal with the original input (Quian Quiroga and Panzeri, 2009) normalized with respect to entropy $\mathcal{MI}_{\mathcal{S}}$, sparsity $\mathcal{HS}$ of the encoded signal (Hoyer, 2004) and spiking efficiency ε (Dupeyroux et al., 2021).

Table S1. Results of the five metrics used for algorithms characterization. Computational complexity is defined through the quantities l (signal length), c (number of channels), n (length of the bitwise representation), w (width of the convolution function).

			$\mathcal{O}(f)$	FSD															
				Butterworth								Gammatone							
				32 Channels				64 Channels				32 Channels				64 Channels			
				$\mathcal{S}$	$\mathcal{MI}_{\mathcal{S}}$	ε	$\mathcal{HS}$	$\mathcal{S}$	$\mathcal{MI}_{\mathcal{S}}$	ε	$\mathcal{HS}$	$\mathcal{S}$	$\mathcal{MI}_{\mathcal{S}}$	ε	$\mathcal{HS}$	$\mathcal{S}$	$\mathcal{MI}_{\mathcal{S}}$	ε	$\mathcal{HS}$
Rate Coding	Poisson Rate		$\mathcal{O}(cl)$	0.5	0.3	0.9	0.8	0.4	0.2	0.9	0.8	1	0.6	0.9	0.7	1	0.6	0.9	0.7
Temporal Coding	Temporal Contrast	TBR	$\mathcal{O}(cl)$	1.2	0.2	0.9	0.7	1.2	0.2	0.9	0.7	1.3	0.4	0.9	0.7	1.3	0.4	0.9	0.7
		SF	$\mathcal{O}(cl)$	0.7	0.3	0.9	0.7	0.7	0.3	0.9	0.7	1	0.5	0.9	0.7	1	0.5	0.9	0.7
		MW	$\mathcal{O}(cl)$	1.6	0.3	0.8	0.5	1.7	0.3	0.8	0.5	1.6	0.6	0.8	0.5	1.6	0.6	0.8	0.5
		ZCSF	$\mathcal{O}(cl)$	0.5	0.3	0.9	0.8	0.5	0.3	0.9	0.8	0.6	0.4	0.9	0.8	0.6	0.4	0.9	0.8
	Filter & Optimizer	HSA	$\mathcal{O}(clw)$	1.4	0.4	0.8	0.6	1.3	0.4	0.8	0.6	1.4	0.6	0.8	0.5	1.4	0.6	0.8	0.5
		MHSA	$\mathcal{O}(clw)$	1.4	0.4	0.8	0.6	1.4	0.4	0.8	0.6	1.4	0.6	0.8	0.5	1.4	0.6	0.8	0.5
		BSA	$\mathcal{O}(clw)$	2	0.4	0.7	0.5	2	0.4	0.7	0.5	1.6	0.6	0.7	0.4	1.6	0.6	0.7	0.4
	Global Referenced	PHASE	$\mathcal{O}(2^n cl)$	0.6	0.4	0.9	0.8	0.5	0.3	0.9	0.8	1.3	0.8	0.9	0.7	1.3	0.8	0.9	0.7
		TTFS	$\mathcal{O}(cln)$	2.3	1.2	0.9	0.7	2.4	1.2	0.9	0.7	2.3	1.5	0.9	0.7	2.3	1.5	0.9	0.7
	Latency/ISI	BURST	$\mathcal{O}(cl)$	1	0.7	0.9	0.8	1	0.6	0.9	0.8	1.3	0.9	0.9	0.8	1.3	0.9	0.9	0.8

			$\mathcal{O}(f)$	WISDM																							
				Butterworth												Gammatone											
				4 Channels				8 Channels				16 Channels				4 Channels				8 Channels				16 Channels			
				$\mathcal{S}$	$\mathcal{MI}_{\mathcal{S}}$	ε	$\mathcal{HS}$	$\mathcal{S}$	$\mathcal{MI}_{\mathcal{S}}$	ε	$\mathcal{HS}$	$\mathcal{S}$	$\mathcal{MI}_{\mathcal{S}}$	ε	$\mathcal{HS}$	$\mathcal{S}$	$\mathcal{MI}_{\mathcal{S}}$	ε	$\mathcal{HS}$	$\mathcal{S}$	$\mathcal{MI}_{\mathcal{S}}$	ε	$\mathcal{HS}$	$\mathcal{S}$	$\mathcal{MI}_{\mathcal{S}}$	ε	$\mathcal{HS}$
Rate Coding	Poisson Rate		$\mathcal{O}(cl)$	0.1	0	0.9	0.9	0.1	0	0.9	0.1	0.3	0	0.9	0.9	0.3	0.1	0.9	0.8	0.3	0.1	0.9	0.8	0.3	0.1	0.9	0.8
Temporal Coding	Temporal Contrast	TBR	$\mathcal{O}(cl)$	2.5	0.3	0.7	0.4	2.4	0.3	0.7	0.4	2.6	0.3	0.7	0.4	2.6	0.2	0.7	0.4	2.6	0.2	0.7	0.4	2.6	0.2	0.7	0.4
		SF	$\mathcal{O}(cl)$	1.1	0.2	0.9	0.7	1.1	0.2	0.9	0.7	1.4	0.2	0.9	0.7	1.4	0.1	0.9	0.7	1.4	0.1	0.9	0.7	1.4	0.1	0.9	0.7
		MW	$\mathcal{O}(cl)$	2.5	0.3	0.7	0.4	2.4	0.3	0.7	0.4	2.6	0.2	0.7	0.4	2.6	0.2	0.7	0.4	2.6	0.2	0.7	0.4	2.6	0.2	0.7	0.4
		ZCSF	$\mathcal{O}(cl)$	1.7	0.5	0.8	0.6	1.8	0.5	0.8	0.6	1.8	0.5	0.8	0.6	1.8	0.7	0.8	0.6	1.8	0.7	0.8	0.6	1.8	0.7	0.8	0.6
	Filter & Optimizer	HSA	$\mathcal{O}(clw)$	1.9	0.4	0.8	0.5	1.7	0.3	0.8	0.5	1.8	0.3	0.8	0.6	1.8	0.5	0.7	0.5	1.8	0.5	0.7	0.5	1.8	0.5	0.7	0.5
		MHSA	$\mathcal{O}(clw)$	3	0.5	0.4	0.2	3.1	0.5	0.4	0.2	2.1	0.5	0.4	0.2	2.1	0.4	0.5	0.3	2.1	0.4	0.5	0.3	2.1	0.4	0.5	0.3
		BSA	$\mathcal{O}(clw)$	2.7	0.2	0.6	0.4	2.8	0.2	0.6	0.4	2.3	0.2	0.6	0.4	2.3	0.4	0.6	0.4	2.3	0.4	0.6	0.4	2.3	0.4	0.6	0.4
	Global Referenced	PHASE	$\mathcal{O}(2^n cl)$	2.1	0.6	0.9	0.6	1.9	0.5	0.8	0.6	2.6	0.4	0.9	0.7	2.6	0.8	0.9	0.6	2.6	0.8	0.8	0.6	2.6	0.8	0.8	0.6
		TTFS	$\mathcal{O}(cln)$	2.4	0.9	0.8	0.6	2.4	0.9	0.8	0.6	2	0.9	0.8	0.6	2	0.9	0.8	0.7	2	0.9	0.9	0.7	2	0.9	0.9	0.7
	Latency/ISI	BURST	$\mathcal{O}(cl)$	1.3	0.6	0.8	0.6	1.1	0.5	0.8	0.6	1.6	0.5	0.8	0.6	1.6	0.9	0.8	0.6	1.6	0.9	0.8	0.	1.6	0.9	0.8	0.6

5 ACCURACY RESULTS

In this section, the best accuracy results for all the explored combinations of network architecture, frequency filtering and encoding technique are reported. All the values are expressed as percentages.

The investigated encoding techniques have been first benchmarked by employing them for CNN-based classification and subsequent transfer learning for spiking CNN models, with the results shown in Section 5.1.1 for FSD data and in Section 5.2.1 for the WISDM dataset. Then, a process of model compression has been carried out implementing two steps: synapse reduction and fine-tuning. The results from the former, allowing to identify the most impactful weights within the spiking CNN models, are summarized in Section 5.1.2 and Section 5.2.2 for the FSD and WISDM datasets respectively. The classification performances achieved after fine-tuning of the compressed CNN and spiking CNN models are instead reported in Section 5.1.3 for FSD data and in Section 5.2.3 for the WISDM dataset.

5.1 Free Spoken Digit (FSD)

5.1.1 Complete Networks

Table S2. Network C6-C12-F2

CNN

Butterworth filter bank		Rate coding		Temporal Contrast								Filter & Oprimizer						Global Referenced				Latency/ISI	
		Poisson		TBR		SF		MW		ZCSF		HSA		MHSA		BSA		PHASE		TTFS		BURST	
		Train	Test	Train	Test	Train	Test	Train	Test	Train	Test	Train	Test	Train	Test	Train	Test	Train	Test	Train	Test	Train	Test
32 Channels	50 Bins	99.0	92.0	99.9	96.0	99.8	95.0	99.8	95.0	99.8	95.0	95.5	83.0	98.8	90.0	98.3	84.0	99.8	94.0	94.5	65.0	99.8	96.0
	250 Bins	99.5	96.0	99.8	96.0	99.8	96.0	99.9	97.0	99.9	96.0	97.8	92.0	97.3	82.0	97.8	84.0	99.9	94.0	94.5	67.0	99.5	94.0
64 Channels	50 Bins	99.8	98.0	99.8	98.0	99.9	98.0	99.9	98.0	99.8	96.0	99.9	80.0	97.8	71.0	99.8	90.0	99.9	97.0	99.3	65.0	99.8	97.0
	125 Bins	99.9	97.0	99.9	98.0	99.9	98.0	99.9	98.0	99.8	97.0	99.5	82.0	97.8	78.0	99.5	87.0	99.9	97.0	98.0	67.0	99.9	92.0

Gammatone filter bank		Rate coding		Temporal Contrast								Filter & Oprimizer						Global Referenced				Latency/ISI	
		Poisson		TBR		SF		MW		ZCSF		HSA		MHSA		BSA		PHASE		TTFS		BURST	
		Train	Test	Train	Test	Train	Test	Train	Test	Train	Test	Train	Test	Train	Test	Train	Test	Train	Test	Train	Test	Train	Test
32 Channels	50 Bins	99.5	90.0	99.5	96.0	99.9	96.0	99.8	90.0	99.9	94.0	94.7	92.0	98.8	91.0	97.0	73.0	99.5	97.0	96.5	77.0	99.3	90.0
	250 Bins	96.7	91.0	99.9	91.0	99.9	94.0	99.3	94.0	99.5	94.0	99.3	95.0	99.3	91.0	92.3	73.0	99.8	95.0	91.8	69.0	98.3	88.0
64 Channels	50 Bins	97.8	91.0	99.9	96.0	99.8	94.0	99.8	93.0	99.8	94.0	99.3	94.0	99.5	97.0	97.5	80.0	99.9	96.0	99.5	81.0	99.5	83.0
	125 Bins	97.0	87.0	99.9	93.0	99.3	93.0	99.5	87.0	99.9	94.0	99.5	98.0	99.8	97.0	97.8	81.0	99.9	97.0	94.7	81.0	99.9	90.0

sCNN

Butterworth filter bank		Rate coding		Temporal Contrast								Filter & Oprimizer						Global Referenced				Latency/ISI	
		Poisson		TBR		SF		MW		ZCSF		HSA		MHSA		BSA		PHASE		TTFS		BURST	
		Train	Test	Train	Test	Train	Test	Train	Test	Train	Test	Train	Test	Train	Test	Train	Test	Train	Test	Train	Test	Train	Test
32 Channels	50 Bins	93.2	82.0	99.8	95.0	99.5	95.0	99.2	93.0	99.2	93.0	89.5	74.0	70.8	60.0	80.0	63.0	98.2	91.0	52.8	29.0	91.5	85.0
	250 Bins	90.2	80.0	96.0	91.0	96.8	83.0	97.5	90.0	98.8	90.0	79.2	69.0	44.0	37.0	61.3	50.0	86.5	74.0	46.0	35.0	96.8	89.0
64 Channels	50 Bins	85.0	67.0	99.2	94.0	99.5	97.0	57.8	38.0	98.5	95.0	88.0	73.0	69.0	52.0	95.8	87.0	93.0	91.0	27.3	15.0	79.2	72.0
	125 Bins	82.5	68.0	99.2	90.0	99.9	98.0	56.8	42.0	90.5	93.0	86.8	70.0	69.8	49.0	98.0	85.0	79.2	75.0	11.2	8.0	64.8	59.0

Gammatone filter bank		Rate coding		Temporal Contrast								Filter & Oprimizer						Global Referenced				Latency/ISI	
		Poisson		TBR		SF		MW		ZCSF		HSA		MHSA		BSA		PHASE		TTFS		BURST	
		Train	Test	Train	Test	Train	Test	Train	Test	Train	Test	Train	Test	Train	Test	Train	Test	Train	Test	Train	Test	Train	Test
32 Channels	50 Bins	96.8	90.0	98.5	92.0	98.2	94.0	83.8	77.0	99.0	91.0	81.5	75.0	78.0	74.0	76.8	64.0	96.2	93.0	69.2	48.0	93.2	84.0
	250 Bins	88.5	86.0	96.8	90.0	94.8	88.0	90.5	80.0	98.0	91.0	72.2	72.0	83.0	82.0	39.5	47.0	90.8	80.0	63.7	55.0	92.0	82.0
64 Channels	50 Bins	95.2	83.0	99.8	94.0	99.0	90.0	90.2	85.0	98.0	92.0	90.5	92.0	96.8	93.0	89.8	75.0	93.5	93.0	74.0	58.0	89.8	81.0
	125 Bins	88.2	81.0	98.2	95.0	93.5	82.0	84.8	79.0	94.0	85.0	99.0	95.0	91.2	87.0	84.5	63.0	95.0	93.0	19.5	18.0	89.8	84.0

Table S3. Network C12-C24-F2

CNN

Butterworth filter bank		Rate coding		Temporal Contrast								Filter & Oprimizer						Global Referenced				Latency/ISI	
		Poisson		TBR		SF		MW		ZCSF		HSA		MHSA		BSA		PHASE		TTFS		BURST	
		Train	Test	Train	Test	Train	Test	Train	Test	Train	Test	Train	Test	Train	Test	Train	Test	Train	Test	Train	Test	Train	Test
32 Channels	50 Bins	99.8	95.0	99.8	97.0	99.9	97.0	99.8	97.0	99.8	96.0	98.5	90.0	99.3	90.0	99.5	90.0	99.8	95.0	98.8	69.0	99.9	96.0
	250 Bins	99.9	92.0	99.8	97.0	99.8	96.0	99.9	96.0	99.9	96.0	98.5	84.0	98.0	89.0	98.0	89.0	99.5	96.0	97.5	67.0	99.5	92.0
64 Channels	50 Bins	99.8	94.0	99.9	96.0	99.9	98.0	99.9	97.0	99.8	97.0	99.0	79.0	98.0	77.0	99.8	84.0	99.8	97.0	99.0	63.0	99.9	97.0
	125 Bins	99.9	97.0	99.9	97.0	99.9	98.0	99.8	96.0	99.9	98.0	97.8	85.0	98.8	80.0	99.9	87.0	99.9	99.0	99.0	63.0	99.8	95.0

Gammatone filter bank		Rate coding		Temporal Contrast								Filter & Oprimizer						Global Referenced				Latency/ISI	
		Poisson		TBR		SF		MW		ZCSF		HSA		MHSA		BSA		PHASE		TTFS		BURST	
		Train	Test	Train	Test	Train	Test	Train	Test	Train	Test	Train	Test	Train	Test	Train	Test	Train	Test	Train	Test	Train	Test
32 Channels	50 Bins	96.5	92.0	99.9	97.0	99.5	92.0	99.8	92.0	99.5	93.0	99.8	95.0	99.3	94.0	97.5	77.0	99.5	95.0	97.5	82.0	99.8	91.0
	250 Bins	98.0	94.0	99.9	95.0	99.5	90.0	99.8	94.0	99.9	93.0	99.9	97.0	98.8	90.0	97.3	82.0	99.9	95.0	99.0	80.0	99.5	92.0
64 Channels	50 Bins	98.5	93.0	99.8	95.0	99.9	93.0	99.5	90.0	99.8	94.0	99.0	97.0	99.8	97.0	98.0	74.0	99.9	97.0	97.0	74.0	99.9	93.0
	125 Bins	98.3	87.0	99.8	97.0	99.3	95.0	99.5	93.0	99.5	95.0	98.8	97.0	99.3	98.0	96.7	78.0	99.9	96.0	98.8	79.0	99.5	91.0

sCNN

Butterworth filter bank		Rate coding		Temporal Contrast								Filter & Oprimizer						Global Referenced				Latency/ISI	
		Poisson		TBR		SF		MW		ZCSF		HSA		MHSA		BSA		PHASE		TTFS		BURST	
		Train	Test	Train	Test	Train	Test	Train	Test	Train	Test	Train	Test	Train	Test	Train	Test	Train	Test	Train	Test	Train	Test
32 Channels	50 Bins	86.8	76.0	99.2	93.0	99.8	96.0	99.5	96.0	95.5	89.0	92.0	73.0	91.8	85.0	84.5	72.0	88.8	81.0	17.8	10.0	56.2	45.0
	250 Bins	97.0	90.0	99.5	97.0	99.8	93.0	99.0	94.0	99.8	96.0	97.0	80.0	85.8	74.0	63.0	53.0	97.0	89.0	44.5	29.0	91.0	80.0
64 Channels	50 Bins	83.5	73.0	99.2	94.0	98.8	95.0	99.2	94.0	95.2	95.0	77.5	58.0	71.2	56.0	97.5	77.0	87.2	84.0	9.0	9.0	74.5	68.0
	125 Bins	76.2	64.0	99.2	94.0	98.8	96.0	90.0	85.0	82.8	77.0	64.8	60.0	83.8	59.0	90.2	78.0	71.0	63.0	10.0	8.0	70.5	60.0

Gammatone filter bank		Rate coding		Temporal Contrast								Filter & Oprimizer						Global Referenced				Latency/ISI	
		Poisson		TBR		SF		MW		ZCSF		HSA		MHSA		BSA		PHASE		TTFS		BURST	
		Train	Test	Train	Test	Train	Test	Train	Test	Train	Test	Train	Test	Train	Test	Train	Test	Train	Test	Train	Test	Train	Test
32 Channels	50 Bins	86.0	77.0	98.2	96.0	97.0	88.0	97.0	87.0	97.2	87.0	96.0	87.0	96.2	89.0	81.2	65.0	88.5	82.0	50.2	41.0	83.2	79.0
	250 Bins	92.0	84.0	98.0	91.0	98.8	92.0	97.5	87.0	98.0	88.0	82.5	78.0	96.2	87.0	78.8	64.0	97.8	91.0	50.2	40.0	91.8	89.0
64 Channels	50 Bins	95.2	89.0	97.2	93.0	98.2	91.0	96.0	91.0	93.5	84.0	99.0	95.0	97.2	90.0	89.5	67.0	92.2	85.0	60.2	49.0	77.0	70.0
	125 Bins	85.5	82.0	95.0	89.0	81.0	72.0	86.2	86.0	91.2	83.0	98.5	94.0	98.8	94.0	92.0	72.0	69.8	73.0	60.0	54.0	70.5	61.0

5.1.2 Synapse reduction

Table S4. Network C6-C12-F2

sCNN

Butterworth filter bank		Complete network								First quartile								Median								Third quartile							
		TBR		SF		MW		ZCSF		TBR		SF		MW		ZCSF		TBR		SF		MW		ZCSF		TBR		SF		MW		ZCSF	
		Train	Test	Train	Test	Train	Test	Train	Test	Train	Test	Train	Test	Train	Test	Train	Test	Train	Test	Train	Test	Train	Test	Train	Test	Train	Test	Train	Test	Train	Test	Train	Test
32 Channels	50 Bins	99.8	95.0	99.5	95.0	99.2	93.0	99.2	93.0	99.8	95.0	99.5	93.0	99.0	93.0	98.5	93.0	97.5	91.0	98.5	94.0	34.2	29.0	95.5	89.0	31.8	32.0	56.2	47.0	93.2	86.0	43.0	41.0
	250 Bins	96.0	91.0	96.8	83.0	97.5	90.0	98.8	90.0	96.8	92.0	95.8	80.0	98.2	89.0	98.0	89.0	82.8	79.0	94.0	78.0	10.0	10.0	81.0	70.0	54.5	50.0	85.5	72.0	10.5	11.0	20.0	16.0
64 Channels	50 Bins	99.2	94.0	99.5	97.0	57.8	38.0	98.5	95.0	99.2	91.0	99.5	97.0	88.5	82.0	97.8	95.0	95.5	86.0	98.8	95.0	10.0	10.0	94.8	94.0	20.8	20.0	60.5	54.0	10.0	10.0	86.0	74.0
	125 Bins	99.2	90.0	99.9	98.0	56.8	42.0	90.5	93.0	97.5	89.0	99.5	97.0	69.8	52.0	89.8	91.0	45.8	42.0	98.5	96.0	10.0	10.0	82.0	69.0	10.2	10.0	37.8	24.0	10.0	10.0	51.5	38.0

Gammatone filter bank		Complete network								First quartile								Median								Third quartile							
		TBR		SF		MW		ZCSF		TBR		SF		MW		ZCSF		TBR		SF		MW		ZCSF		TBR		SF		MW		ZCSF	
		Train	Test	Train	Test	Train	Test	Train	Test	Train	Test	Train	Test	Train	Test	Train	Test	Train	Test	Train	Test	Train	Test	Train	Test	Train	Test	Train	Test	Train	Test	Train	Test
32 Channels	50 Bins	98.5	92.0	98.2	94.0	83.8	77.0	99.0	91.0	98.5	92.0	96.5	92.0	88.8	78.0	98.8	90.0	94.5	90.0	84.5	77.0	52.0	45.0	97.8	87.0	48.8	48.0	91.5	89.0	10.0	10.0	14.2	15.0
	250 Bins	96.8	90.0	94.8	88.0	90.5	80.0	98.0	91.0	97.2	91.0	94.2	89.0	84.5	76.0	99.2	89.0	90.5	86.0	84.2	82.0	11.0	10.0	98.2	90.0	63.2	61.0	33.2	34.0	10.0	10.0	94.2	89.0
64 Channels	50 Bins	99.8	94.0	99.0	90.0	90.2	85.0	98.0	92.0	99.5	94.0	99.0	90.0	80.5	77.0	98.0	92.0	97.5	91.0	98.2	90.0	19.2	19.0	96.5	92.0	93.8	85.0	90.0	82.0	11.2	12.0	83.5	76.0
	125 Bins	98.2	95.0	93.5	82.0	84.8	79.0	94.0	85.0	98.2	96.0	93.2	84.0	71.8	65.0	92.8	85.0	85.0	80.0	83.2	81.0	22.0	17.0	92.0	82.0	33.0	30.0	46.8	49.0	9.8	10.0	81.8	68.0

Table S5. Network C12-C24-F2

sCNN

Butterworth filter bank		Complete network								First quartile								Median								Third quartile							
		TBR		SF		MW		ZCSF		TBR		SF		MW		ZCSF		TBR		SF		MW		ZCSF		TBR		SF		MW		ZCSF	
		Train	Test	Train	Test	Train	Test	Train	Test	Train	Test	Train	Test	Train	Test	Train	Test	Train	Test	Train	Test	Train	Test	Train	Test	Train	Test	Train	Test	Train	Test	Train	Test
32 Channels	50 Bins	99.2	93.0	99.8	96.0	99.5	96.0	95.5	89.0	99.0	96.0	99.5	96.0	99.2	95.0	96.0	88.0	93.5	84.0	98.2	94.0	17.5	15.0	94.8	90.0	18.8	25.0	45.8	35.0	10.0	10.0	45.5	35.0
	250 Bins	99.5	97.0	99.8	93.0	99.0	94.0	99.8	96.0	99.2	97.0	99.8	91.0	95.0	84.0	99.8	96.0	97.2	93.0	96.2	91.0	10.2	10.0	99.5	96.0	49.2	49.0	46.2	36.0	10.0	10.0	90.2	85.0
64 Channels	50 Bins	99.2	94.0	98.8	95.0	99.2	94.0	95.2	95.0	99.8	93.0	98.8	96.0	98.5	90.0	94.5	93.0	99.0	93.0	96.5	91.0	97.0	94.0	93.0	90.0	37.0	40.0	47.2	40.0	10.0	10.0	68.5	65.0
	125 Bins	99.2	94.0	98.8	96.0	90.0	85.0	82.8	77.0	99.0	93.0	98.8	96.0	91.2	86.0	82.0	78.0	97.2	91.0	92.8	92.0	28.2	20.0	79.5	73.0	49.8	37.0	53.5	41.0	6.8	6.0	66.2	63.0

Gammatone filter bank		Complete network								First quartile								Median								Third quartile							
		TBR		SF		MW		ZCSF		TBR		SF		MW		ZCSF		TBR		SF		MW		ZCSF		TBR		SF		MW		ZCSF	
		Train	Test	Train	Test	Train	Test	Train	Test	Train	Test	Train	Test	Train	Test	Train	Test	Train	Test	Train	Test	Train	Test	Train	Test	Train	Test	Train	Test	Train	Test	Train	Test
32 Channels	50 Bins	98.2	96.0	97.0	88.0	97.0	87.0	97.2	87.0	97.8	95.0	96.0	87.0	96.5	86.0	97.2	86.0	65.5	66.0	92.0	83.0	94.2	85.0	88.8	83.0	18.0	16.0	57.5	46.0	49.5	38.0	70.2	69.0
	250 Bins	98.0	91.0	98.8	92.0	97.5	87.0	98.0	88.0	92.0	83.0	98.5	92.0	91.0	78.0	98.5	90.0	65.0	69.0	97.0	94.0	86.5	81.0	97.8	90.0	20.5	24.0	47.5	44.0	41.0	38.0	70.0	69.0
64 Channels	50 Bins	97.2	93.0	98.2	91.0	96.0	91.0	93.5	84.0	96.0	91.0	98.0	91.0	71.8	70.0	94.2	84.0	95.2	90.0	94.5	86.0	13.2	10.0	88.2	82.0	61.0	63.0	52.8	49.0	9.2	9.0	71.0	68.0
	125 Bins	95.0	89.0	81.0	72.0	86.2	86.0	91.2	83.0	95.0	89.0	78.8	67.0	37.5	35.0	90.0	83.0	84.8	78.0	70.2	59.0	7.8	8.0	86.5	82.0	16.0	14.0	24.0	23.0	6.5	6.0	68.5	65.0

5.1.3 Fine tuning

Table S6. Network C6-C12-F2

CNN

Butterworth filter bank		Above median								Upper quartile							
		TBR		SF		MW		ZCSF		TBR		SF		MW		ZCSF	
		Train	Test	Train	Test	Train	Test	Train	Test	Train	Test	Train	Test	Train	Test	Train	Test
32 Channels	50 Bins	99.9	96.0	99.9	95.0	99.9	97.0	99.9	96.0	99.9	96.0	99.9	95.0	99.8	95.0	99.8	96.0
	250 Bins	99.9	96.0	99.8	96.0	99.9	98.0	99.9	97.0	99.9	96.0	99.8	95.0	99.5	98.0	99.9	97.0
64 Channels	50 Bins	99.9	98.0	99.9	99.9	99.9	98.0	99.9	97.0	99.9	96.0	99.9	97.0	99.9	98.0	99.9	97.0
	125 Bins	99.8	98.0	99.9	98.0	99.9	97.0	99.9	98.0	99.8	97.0	99.9	99.0	99.8	98.0	99.9	99.0

Gammatone filter bank		Above median								Upper quartile							
		TBR		SF		MW		ZCSF		TBR		SF		MW		ZCSF	
		Train	Test	Train	Test	Train	Test	Train	Test	Train	Test	Train	Test	Train	Test	Train	Test
32 Channels	50 Bins	99.9	95.0	99.9	96.0	99.9	89.0	99.9	95.0	99.9	95.0	99.8	96.0	99.5	88.0	99.9	94.0
	250 Bins	99.9	92.0	99.9	91.0	99.8	92.0	99.9	95.0	99.9	93.0	99.9	94.0	99.3	94.0	99.5	95.0
64 Channels	50 Bins	99.9	96.0	99.9	95.0	99.9	92.0	99.8	94.0	99.9	97.0	99.8	96.0	99.5	94.0	99.9	94.0
	125 Bins	99.8	95.0	99.9	94.0	99.9	94.0	99.9	93.0	99.9	93.0	99.8	94.0	99.3	95.0	99.9	94.0

sCNN

Butterworth filter bank		Above median								Upper quartile							
		TBR		SF		MW		ZCSF		TBR		SF		MW		ZCSF	
		Train	Test	Train	Test	Train	Test	Train	Test	Train	Test	Train	Test	Train	Test	Train	Test
32 Channels	50 Bins	99.8	95.0	99.9	95.0	99.0	94.0	99.9	96.0	99.2	94.0	99.9	94.0	99.2	94.0	99.0	95.0
	250 Bins	98.0	94.0	92.8	78.0	99.2	90.0	99.2	89.0	93.0	89.0	93.5	77.0	99.2	96.0	99.9	94.0
64 Channels	50 Bins	99.5	94.0	99.8	98.0	97.8	94.0	99.2	95.0	99.8	94.0	99.9	96.0	99.8	96.0	99.8	97.0
	125 Bins	99.2	94.0	99.9	98.0	86.8	78.0	94.0	93.0	99.2	94.0	99.5	99.0	96.0	90.0	96.2	97.0

Gammatone filter bank		Above median								Upper quartile							
		TBR		SF		MW		ZCSF		TBR		SF		MW		ZCSF	
		Train	Test	Train	Test	Train	Test	Train	Test	Train	Test	Train	Test	Train	Test	Train	Test
32 Channels	50 Bins	99.2	94.0	99.2	94.0	92.5	77.0	99.2	93.0	98.0	93.0	98.8	94.0	86.2	77.0	97.2	85.0
	250 Bins	96.5	90.0	88.2	82.0	97.0	86.0	94.2	88.0	86.8	83.0	87.5	85.0	95.5	86.0	97.8	92.0
64 Channels	50 Bins	99.9	96.0	99.2	91.0	95.8	84.0	99.0	92.0	99.5	97.0	98.8	90.0	97.5	93.0	98.0	91.0
	125 Bins	98.0	94.0	96.2	88.0	95.0	90.0	96.5	86.0	98.0	95.0	91.0	81.0	87.5	89.0	93.2	84.0

Table S7. Network C12-C24-F2

CNN

Butterworth filter bank		Above median								Upper quartile							
		TBR		SF		MW		ZCSF		TBR		SF		MW		ZCSF	
		Train	Test	Train	Test	Train	Test	Train	Test	Train	Test	Train	Test	Train	Test	Train	Test
32 Channels	50 Bins	99.9	95.0	99.9	96.0	99.9	96.0	99.9	96.0	99.8	95.0	99.9	97.0	98.8	92.0	99.9	96.0
	250 Bins	99.9	95.0	99.9	97.0	99.9	96.0	99.9	96.0	99.9	96.0	99.5	96.0	99.8	92.0	99.9	97.0
64 Channels	50 Bins	99.9	97.0	99.9	99.0	99.9	98.0	99.9	97.0	99.9	97.0	99.9	98.0	99.5	98.0	99.9	97.0
	125 Bins	99.9	97.0	99.9	98.0	99.9	96.0	99.9	97.0	99.8	97.0	99.9	98.0	99.0	89.0	99.9	98.0

Gammatone filter bank		Above median								Upper quartile							
		TBR		SF		MW		ZCSF		TBR		SF		MW		ZCSF	
		Train	Test	Train	Test	Train	Test	Train	Test	Train	Test	Train	Test	Train	Test	Train	Test
32 Channels	50 Bins	99.9	97.0	99.9	90.0	99.9	93.0	99.9	94.0	99.8	95.0	99.8	90.0	99.8	91.0	99.8	93.0
	250 Bins	99.9	94.0	99.9	91.0	99.9	93.0	99.9	94.0	99.9	95.0	99.8	93.0	99.8	89.0	99.9	94.0
64 Channels	50 Bins	99.8	96.0	99.9	94.0	99.3	93.0	99.9	93.0	99.9	96.0	99.9	92.0	97.8	89.0	99.5	93.0
	125 Bins	99.8	97.0	99.9	94.0	99.8	93.0	99.9	95.0	99.9	97.0	99.8	94.0	99.5	92.0	99.5	94.0

sCNN

Butterworth filter bank		Above median								Upper quartile							
		TBR		SF		MW		ZCSF		TBR		SF		MW		ZCSF	
		Train	Test	Train	Test	Train	Test	Train	Test	Train	Test	Train	Test	Train	Test	Train	Test
32 Channels	50 Bins	99.8	94.0	99.9	97.0	99.0	95.0	98.5	93.0	98.0	89.0	99.2	95.0	97.8	88.0	88.0	83.0
	250 Bins	99.8	97.0	99.9	95.0	98.5	93.0	99.9	95.0	99.5	95.0	99.0	91.0	97.5	90.0	99.9	94.0
64 Channels	50 Bins	99.5	94.0	99.0	96.0	97.8	91.0	98.0	95.0	98.0	93.0	95.5	92.0	93.2	87.0	94.2	94.0
	125 Bins	99.9	95.0	99.0	98.0	82.8	69.0	89.0	84.0	98.0	91.0	93.0	88.0	58.5	46.0	73.8	67.0

Gammatone filter bank		Above median								Upper quartile							
		TBR		SF		MW		ZCSF		TBR		SF		MW		ZCSF	
		Train	Test	Train	Test	Train	Test	Train	Test	Train	Test	Train	Test	Train	Test	Train	Test
32 Channels	50 Bins	98.8	95.0	96.8	87.0	99.0	92.0	96.5	87.0	92.5	91.0	91.8	79.0	97.2	91.0	94.0	83.0
	250 Bins	98.2	91.0	98.8	92.0	99.0	89.0	98.0	89.0	96.2	94.0	95.8	92.0	96.2	89.0	94.5	85.0
64 Channels	50 Bins	97.2	94.0	95.8	88.0	90.0	90.0	88.5	82.0	93.8	90.0	91.2	78.0	67.2	59.0	80.8	71.0
	125 Bins	95.0	90.0	78.8	66.0	72.5	72.0	81.0	75.0	82.2	74.0	55.0	45.0	63.2	61.0	82.8	79.0

5.2 Wireless Sensor Data Mining (WISDM)

5.2.1 Complete Networks

Table S8. Network C6-C12-F2

CNN

Butterworth filter bank		Rate coding		Temporal Contrast								Filter & Oprimizer						Global Referenced				Latency/ISI	
		Poisson		TBR		SF		MW		ZCSF		HSA		MHSA		BSA		PHASE		TTFS		BURST	
		Train	Test	Train	Test	Train	Test	Train	Test	Train	Test	Train	Test	Train	Test	Train	Test	Train	Test	Train	Test	Train	Test
4 Channels	24 Bins	97.1	71.7	95.4	83.3	96.7	73.3	91.7	91.7	96.7	85.0	92.5	73.3	92.1	81.7	89.2	85.0	97.1	80.0	90.8	75.0	95.0	76.7
8 Channels	18 Bins	97.9	75.0	98.8	88.3	97.1	83.3	82.5	83.3	99.2	83.3	94.2	85.0	95.8	85.0	95.0	81.7	98.8	88.3	70.0	56.7	94.2	83.3
16 Channels	18 Bins	97.5	81.7	99.2	83.3	97.5	90.0	89.2	76.7	99.9	91.7	92.9	85.0	97.5	91.7	97.9	93.3	99.6	90.0	56.7	55.0	92.9	81.7

Gammatone filter bank		Rate coding		Temporal Contrast								Filter & Oprimizer						Global Referenced				Latency/ISI	
		Poisson		TBR		SF		MW		ZCSF		HSA		MHSA		BSA		PHASE		TTFS		BURST	
		Train	Test	Train	Test	Train	Test	Train	Test	Train	Test	Train	Test	Train	Test	Train	Test	Train	Test	Train	Test	Train	Test
4 Channels	24 Bins	93.3	73.3	95.8	70.0	95.4	66.7	90.4	66.7	95.8	60.0	95.4	75.0	94.6	70.0	91.3	61.7	94.2	63.3	91.3	61.7	91.3	61.7
8 Channels	18 Bins	95.0	73.3	88.3	65.0	95.8	68.3	90.0	63.3	97.1	68.3	95.8	71.7	96.2	81.7	96.2	70.0	95.8	68.3	90.0	68.3	96.2	58.3
16 Channels	18 Bins	95.8	76.7	90.8	68.3	98.8	60.0	82.1	61.7	96.7	63.3	96.7	66.7	97.9	68.3	95.8	71.7	96.7	70.0	95.8	61.7	98.3	71.7

sCNN

Butterworth filter bank		Rate coding		Temporal Contrast								Filter & Oprimizer						Global Referenced				Latency/ISI	
		Poisson		TBR		SF		MW		ZCSF		HSA		MHSA		BSA		PHASE		TTFS		BURST	
		Train	Test	Train	Test	Train	Test	Train	Test	Train	Test	Train	Test	Train	Test	Train	Test	Train	Test	Train	Test	Train	Test
4 Channels	24 Bins	17.1	16.7	37.9	33.3	80.4	63.3	35.4	28.3	92.5	68.3	80.8	73.3	39.2	35.0	36.2	26.7	80.8	66.7	45.4	36.7	22.1	20.0
8 Channels	18 Bins	10.4	5.0	57.5	58.3	90.0	75.0	22.9	21.7	95.0	83.3	76.2	81.7	57.5	58.3	39.6	38.3	82.5	71.7	22.5	23.3	61.3	58.3
16 Channels	18 Bins	17.5	13.3	36.2	31.7	97.1	88.3	18.8	18.3	97.1	93.3	80.0	86.7	64.6	65.0	58.8	53.3	92.1	75.0	28.7	28.3	63.3	68.3

Gammatone filter bank		Rate coding		Temporal Contrast								Filter & Oprimizer						Global Referenced				Latency/ISI	
		Poisson		TBR		SF		MW		ZCSF		HSA		MHSA		BSA		PHASE		TTFS		BURST	
		Train	Test	Train	Test	Train	Test	Train	Test	Train	Test	Train	Test	Train	Test	Train	Test	Train	Test	Train	Test	Train	Test
4 Channels	24 Bins	52.9	48.3	24.6	21.7	59.6	36.7	38.8	31.7	82.1	61.7	58.3	45.0	42.9	40.0	71.7	53.3	82.1	61.7	61.7	45.0	76.2	63.3
8 Channels	18 Bins	66.2	56.7	19.6	15.0	35.4	25.0	29.2	26.7	49.2	46.7	50.8	50.0	47.5	45.0	30.4	28.3	43.3	43.3	60.8	48.3	55.8	50.0
16 Channels	18 Bins	71.7	56.7	33.8	35.0	36.2	21.7	26.2	21.7	46.2	43.3	43.3	41.7	53.8	55.0	30.0	28.3	39.2	41.7	27.9	26.7	59.2	48.3

Table S9. Network C12-C24-F2

CNN

Butterworth filter bank		Rate coding		Temporal Contrast								Filter & Oprimizer						Global Referenced				Latency/ISI	
		Poisson		TBR		SF		MW		ZCSF		HSA		MHSA		BSA		PHASE		TTFS		BURST	
		Train	Test	Train	Test	Train	Test	Train	Test	Train	Test	Train	Test	Train	Test	Train	Test	Train	Test	Train	Test	Train	Test
4 Channels	24 Bins	97.5	66.7	97.1	85.0	96.7	73.3	98.8	81.7	98.3	85.0	97.1	80.0	97.9	86.7	96.7	85.0	95.8	68.3	92.9	81.7	97.5	83.3
8 Channels	18 Bins	99.2	73.3	97.1	88.3	98.8	83.3	93.3	83.3	98.3	86.7	96.2	90.0	95.8	86.7	98.3	88.3	97.9	85.0	79.2	63.3	93.8	80.0
16 Channels	18 Bins	96.7	83.3	98.8	88.3	99.2	90.0	86.3	78.3	98.3	95.0	94.2	85.0	97.5	95.0	98.3	93.3	97.9	88.3	16.7	16.7	95.0	81.7

Gammatone filter bank		Rate coding		Temporal Contrast								Filter & Oprimizer						Global Referenced				Latency/ISI	
		Poisson		TBR		SF		MW		ZCSF		HSA		MHSA		BSA		PHASE		TTFS		BURST	
		Train	Test	Train	Test	Train	Test	Train	Test	Train	Test	Train	Test	Train	Test	Train	Test	Train	Test	Train	Test	Train	Test
4 Channels	24 Bins	95.8	78.3	95.4	61.7	97.1	73.3	95.0	68.3	96.7	65.0	96.7	66.7	95.8	76.7	92.9	75.0	96.2	66.7	95.8	63.3	96.2	70.0
8 Channels	18 Bins	96.2	78.3	92.1	68.3	94.6	75.0	93.3	63.3	98.8	66.7	97.5	75.0	90.8	75.0	95.0	71.7	96.7	61.7	90.8	65.0	97.5	68.3
16 Channels	18 Bins	96.7	75.0	94.2	60.0	97.5	70.0	89.6	65.0	96.2	63.3	97.1	75.0	96.2	76.7	95.0	63.3	95.8	73.3	96.2	60.0	95.8	73.3

sCNN

Butterworth filter bank		Rate coding		Temporal Contrast								Filter & Oprimizer						Global Referenced				Latency/ISI	
		Poisson		TBR		SF		MW		ZCSF		HSA		MHSA		BSA		PHASE		TTFS		BURST	
		Train	Test	Train	Test	Train	Test	Train	Test	Train	Test	Train	Test	Train	Test	Train	Test	Train	Test	Train	Test	Train	Test
4 Channels	24 Bins	18.3	16.7	80.4	68.3	87.1	66.7	57.9	48.3	92.5	80.0	75.4	63.3	56.2	53.3	70.8	76.7	82.5	60.0	44.6	40.0	18.3	16.7
8 Channels	18 Bins	17.1	10.0	60.4	60.0	84.6	71.7	19.6	16.7	96.7	86.7	90.8	85.0	69.6	68.3	91.7	80.0	92.5	78.3	25.4	28.3	60.8	58.3
16 Channels	18 Bins	16.7	16.7	83.3	71.7	95.8	86.7	19.6	16.7	97.1	90.0	94.6	85.0	71.7	71.7	57.5	60.0	96.7	86.7	16.7	16.7	87.5	88.3

Gammatone filter bank		Rate coding		Temporal Contrast								Filter & Oprimizer						Global Referenced				Latency/ISI	
		Poisson		TBR		SF		MW		ZCSF		HSA		MHSA		BSA		PHASE		TTFS		BURST	
		Train	Test	Train	Test	Train	Test	Train	Test	Train	Test	Train	Test	Train	Test	Train	Test	Train	Test	Train	Test	Train	Test
4 Channels	24 Bins	37.5	26.7	45.4	41.7	67.1	53.3	50.0	45.0	87.1	61.7	72.5	53.3	54.2	48.3	37.5	31.7	86.7	61.7	75.0	55.0	74.6	66.7
8 Channels	18 Bins	60.4	56.7	40.4	38.3	53.8	36.7	28.7	20.0	62.9	56.7	48.3	40.0	52.9	48.3	30.8	28.3	70.8	56.7	62.1	48.3	67.1	51.7
16 Channels	18 Bins	40.0	35.0	47.5	43.3	55.4	46.7	35.0	21.7	64.2	50.0	31.2	26.7	65.4	58.3	31.7	31.7	50.8	46.7	46.7	40.0	68.3	50.0

5.2.2 Synapse reduction

Table S10. Network C6-C12-F2

sCNN

Butterworth filter bank		Complete network								First quartile							
		TBR		SF		MW		ZCSF		TBR		SF		MW		ZCSF	
		Train	Test	Train	Test	Train	Test	Train	Test	Train	Test	Train	Test	Train	Test	Train	Test
4 Channels	24 Bins	37.9	33.3	80.4	63.3	35.4	28.3	92.5	68.3	27.5	23.3	78.3	65.0	32.5	30.0	89.6	76.7
8 Channels	18 Bins	57.5	58.3	90.0	75.0	22.9	21.7	95.0	83.3	62.1	63.3	90.4	75.0	27.9	30.0	94.2	85.0
16 Channels	18 Bins	36.2	31.7	97.1	88.3	18.8	18.3	97.1	93.3	32.5	30.0	96.7	86.7	20.4	18.3	97.9	93.3

Butterworth filter bank		Median								Third quartile							
		TBR		SF		MW		ZCSF		TBR		SF		MW		ZCSF	
		Train	Test	Train	Test	Train	Test	Train	Test	Train	Test	Train	Test	Train	Test	Train	Test
4 Channels	24 Bins	24.6	25.0	66.7	53.3	17.5	18.3	81.2	68.3	16.7	16.7	30.8	25.0	16.7	16.7	36.2	41.7
8 Channels	18 Bins	20.8	23.3	85.8	76.7	24.2	21.7	82.9	76.7	20.0	23.3	71.7	70.0	16.7	16.7	62.1	56.7
16 Channels	18 Bins	29.2	21.7	93.3	85.0	16.7	16.7	67.9	68.3	20.0	18.3	68.8	65.0	16.7	16.7	25.4	20.0

Gammatone filter bank		Complete network								First quartile							
		ZCSF		BSA		PHASE		BURST		ZCSF		BSA		PHASE		BURST	
		Train	Test	Train	Test	Train	Test	Train	Test	Train	Test	Train	Test	Train	Test	Train	Test
4 Channels	24 Bins	82.1	61.7	71.7	53.3	82.1	61.7	76.2	63.3	82.1	63.3	65.8	50.0	83.3	63.3	77.9	63.3
8 Channels	18 Bins	49.2	46.7	30.4	28.3	43.3	43.3	55.8	50.0	46.7	43.3	30.4	28.3	45.0	43.3	43.8	40.0
16 Channels	18 Bins	46.2	43.3	30.0	28.3	39.2	41.7	59.2	48.3	44.6	45.0	30.0	30.0	40.0	41.7	62.1	50.0

Gammatone filter bank		Median								Third quartile							
		ZCSF		BSA		PHASE		BURST		ZCSF		BSA		PHASE		BURST	
		Train	Test	Train	Test	Train	Test	Train	Test	Train	Test	Train	Test	Train	Test	Train	Test
4 Channels	24 Bins	72.1	58.3	56.2	43.3	77.1	63.3	57.5	38.3	36.7	26.7	35.8	21.7	38.3	35.0	29.6	23.3
8 Channels	18 Bins	42.9	38.3	17.1	16.7	52.9	45.0	34.6	28.3	22.1	21.7	16.7	16.7	21.2	21.7	25.8	23.3
16 Channels	18 Bins	41.2	40.0	17.1	18.3	31.7	26.7	56.7	50.0	27.1	25.0	16.7	16.7	25.0	20.0	22.9	21.7

Table S11. Network C12-C24-F2

sCNN

Butterworth filter bank		Complete network								First quartile							
		TBR		SF		MW		ZCSF		TBR		SF		MW		ZCSF	
		Train	Test	Train	Test	Train	Test	Train	Test	Train	Test	Train	Test	Train	Test	Train	Test
4 Channels	24 Bins	80.4	68.3	87.1	66.7	57.9	48.3	92.5	80.0	58.8	48.3	86.2	66.7	73.3	70.0	94.2	80.0
8 Channels	18 Bins	60.4	60.0	84.6	71.7	19.6	16.7	96.7	86.7	64.2	63.3	83.8	75.0	24.6	25.0	97.1	85.0
16 Channels	18 Bins	83.3	71.7	95.8	86.7	19.6	16.7	97.1	90.0	71.2	60.0	93.3	83.3	17.5	16.7	96.2	90.0

Butterworth filter bank		Median								Third quartile							
		TBR		SF		MW		ZCSF		TBR		SF		MW		ZCSF	
		Train	Test	Train	Test	Train	Test	Train	Test	Train	Test	Train	Test	Train	Test	Train	Test
4 Channels	24 Bins	48.8	40.0	82.1	65.0	27.9	26.7	81.7	70.0	24.2	20.0	53.8	36.7	17.5	18.3	31.7	30.0
8 Channels	18 Bins	17.9	16.7	83.8	71.7	16.7	16.7	91.7	80.0	17.1	16.7	75.8	66.7	16.7	16.7	60.8	51.7
16 Channels	18 Bins	31.2	28.3	90.4	80.0	21.2	18.3	94.6	81.7	32.9	28.3	57.5	53.3	16.7	18.3	72.1	66.7

Gammatone filter bank		Complete network								First quartile							
		ZCSF		BSA		PHASE		BURST		ZCSF		BSA		PHASE		BURST	
		Train	Test	Train	Test	Train	Test	Train	Test	Train	Test	Train	Test	Train	Test	Train	Test
4 Channels	24 Bins	87.1	61.7	37.5	31.7	86.7	61.7	74.6	66.7	82.9	61.7	29.6	28.3	86.7	63.3	67.9	56.7
8 Channels	18 Bins	62.9	56.7	30.8	28.3	70.8	56.7	67.1	51.7	62.5	56.7	31.2	26.7	72.5	55.0	67.9	53.3
16 Channels	18 Bins	64.2	50.0	31.7	31.7	50.8	46.7	68.3	50.0	47.9	40.0	30.4	31.7	47.9	45.0	68.3	50.0

Gammatone filter bank		Median								Third quartile							
		ZCSF		BSA		PHASE		BURST		ZCSF		BSA		PHASE		BURST	
		Train	Test	Train	Test	Train	Test	Train	Test	Train	Test	Train	Test	Train	Test	Train	Test
4 Channels	24 Bins	76.2	58.3	18.8	16.7	84.6	61.7	52.5	45.0	58.3	48.3	16.7	16.7	50.4	48.3	31.7	31.7
8 Channels	18 Bins	50.0	43.3	27.9	21.7	44.6	41.7	54.2	51.7	36.2	36.7	14.6	13.3	25.0	23.3	20.4	21.7
16 Channels	18 Bins	32.1	31.7	29.6	28.3	18.3	21.7	58.8	51.7	17.5	20.0	22.1	20.0	15.0	16.7	25.0	21.7

5.2.3 Fine tuning

Table S12. Network C6-C12-F2

CNN

Butterworth filter bank		Above median								Upper quartile							
		SF		ZCSF		PHASE		BURST		SF		ZCSF		PHASE		BURST	
		Train	Test	Train	Test	Train	Test	Train	Test	Train	Test	Train	Test	Train	Test	Train	Test
4 Channels	24 Bins	98.8	76.7	97.9	86.7	98.8	80.0	94.6	81.7	92.9	73.3	90.8	78.3	83.7	68.3	90.0	76.7
8 Channels	18 Bins	98.8	83.3	99.6	85.0	99.2	86.7	94.2	86.7	96.7	83.3	97.9	85.0	97.1	88.3	92.5	83.3
16 Channels	18 Bins	99.9	90.0	99.9	93.3	99.9	88.3	93.3	83.3	99.2	90.0	99.6	91.7	99.6	88.3	91.7	85.0

Gammatone filter bank		Above median								Upper quartile							
		ZCSF		TTFS		PHASE		BURST		ZCSF		TTFS		PHASE		BURST	
		Train	Test	Train	Test	Train	Test	Train	Test	Train	Test	Train	Test	Train	Test	Train	Test
4 Channels	24 Bins	98.8	68.3	96.2	61.7	97.5	63.3	95.8	68.3	96.2	65.0	90.4	68.3	97.5	63.3	89.2	65.0
8 Channels	18 Bins	99.2	63.3	92.1	68.3	99.6	66.7	98.3	65.0	82.5	61.7	89.2	63.3	85.4	65.0	93.8	66.7
16 Channels	18 Bins	98.8	68.3	95.8	65.0	98.8	71.7	98.8	73.3	96.7	63.3	63.3	51.7	77.5	66.7	80.8	70.0

sCNN

Butterworth filter bank		Above median								Upper quartile							
		SF		ZCSF		PHASE		BURST		SF		ZCSF		PHASE		BURST	
		Train	Test	Train	Test	Train	Test	Train	Test	Train	Test	Train	Test	Train	Test	Train	Test
4 Channels	24 Bins	80.0	61.7	92.9	76.7	82.1	71.7	28.7	25.0	72.9	58.3	68.8	60.0	60.0	56.7	44.2	40.0
8 Channels	18 Bins	92.5	78.3	92.5	81.7	85.4	75.0	62.1	56.7	90.0	75.0	96.2	88.3	93.8	83.3	68.3	66.7
16 Channels	18 Bins	97.1	86.7	97.5	93.3	95.8	80.0	85.4	81.7	96.2	85.0	99.2	91.7	97.5	85.0	79.6	68.3

Gammatone filter bank		Above median								Upper quartile							
		ZCSF		TTFS		PHASE		BURST		ZCSF		TTFS		PHASE		BURST	
		Train	Test	Train	Test	Train	Test	Train	Test	Train	Test	Train	Test	Train	Test	Train	Test
4 Channels	24 Bins	73.8	60.0	66.7	46.7	82.1	65.0	82.5	68.3	68.3	53.3	72.9	51.7	85.0	65.0	77.1	61.7
8 Channels	18 Bins	58.3	48.3	71.7	58.3	45.0	40.0	63.3	51.7	48.8	33.3	71.7	53.3	49.6	48.3	57.9	53.3
16 Channels	18 Bins	55.4	48.3	32.1	33.3	39.6	41.7	60.0	50.0	57.1	56.7	23.8	20.0	52.1	45.0	70.0	61.7

Table S13. Network C12-C24-F2

CNN

Butterworth filter bank		Above median								Upper quartile							
		SF		ZCSF		PHASE		BURST		SF		ZCSF		PHASE		BURST	
		Train	Test	Train	Test	Train	Test	Train	Test	Train	Test	Train	Test	Train	Test	Train	Test
4 Channels	24 Bins	98.8	78.3	99.2	81.7	99.6	76.7	98.3	83.3	98.3	78.3	95.4	86.7	89.6	70.0	96.7	83.3
8 Channels	18 Bins	99.6	81.7	99.6	88.3	99.2	85.0	97.5	81.7	98.3	85.0	98.3	88.3	97.9	86.7	96.7	83.3
16 Channels	18 Bins	99.9	93.3	99.9	96.7	99.9	88.3	97.1	80.0	99.2	93.3	99.6	95.0	99.6	88.3	96.7	86.7

Gammatone filter bank		Above median								Upper quartile							
		ZCSF		TTFS		PHASE		BURST		ZCSF		TTFS		PHASE		BURST	
		Train	Test	Train	Test	Train	Test	Train	Test	Train	Test	Train	Test	Train	Test	Train	Test
4 Channels	24 Bins	98.3	65.0	99.2	66.7	98.8	65.0	97.1	66.7	97.1	65.0	94.2	75.0	98.3	65.0	92.9	71.7
8 Channels	18 Bins	99.2	63.3	97.5	70.0	99.6	68.3	99.2	65.0	82.9	63.3	79.6	63.3	92.9	61.7	96.2	73.3
16 Channels	18 Bins	98.3	60.0	99.2	63.3	98.3	70.0	99.9	71.7	97.5	66.7	93.8	63.3	86.3	66.7	98.3	71.7

sCNN

Butterworth filter bank		Above median								Upper quartile							
		SF		ZCSF		PHASE		BURST		SF		ZCSF		PHASE		BURST	
		Train	Test	Train	Test	Train	Test	Train	Test	Train	Test	Train	Test	Train	Test	Train	Test
4 Channels	24 Bins	88.3	70.0	90.8	83.3	73.8	56.7	20.0	16.7	74.6	60.0	68.3	60.0	52.5	43.3	27.5	26.7
8 Channels	18 Bins	83.3	70.0	91.7	80.0	95.0	85.0	68.3	66.7	94.2	85.0	96.7	85.0	89.2	80.0	71.2	66.7
16 Channels	18 Bins	97.1	95.0	97.5	88.3	98.3	90.0	86.2	88.3	98.3	91.7	99.2	91.7	97.9	88.3	73.8	75.0

Gammatone filter bank		Above median								Upper quartile							
		ZCSF		TTFS		PHASE		BURST		ZCSF		TTFS		PHASE		BURST	
		Train	Test	Train	Test	Train	Test	Train	Test	Train	Test	Train	Test	Train	Test	Train	Test
4 Channels	24 Bins	86.2	61.7	75.4	56.7	85.8	60.0	72.9	61.7	76.7	56.7	68.3	56.7	84.2	56.7	58.8	50.0
8 Channels	18 Bins	66.7	53.3	63.3	48.3	57.9	50.0	67.5	51.7	54.6	56.7	35.8	21.7	50.0	41.7	65.0	51.7
16 Channels	18 Bins	75.4	60.0	49.6	35.0	57.9	56.7	72.1	58.3	66.7	55.0	36.2	35.0	47.5	36.7	62.5	51.7

REFERENCES

Aertsen, A. M. H. J. and Johannesma, P. I. M. (1980). Spectro-temporal receptive fields of auditory neurons in the grassfrog. *Biological Cybernetics* 38, 223–234. doi:10.1007/BF00337015

Dupeyroux, J., Stroobants, S., and de Croon, G. (2021). A toolbox for neuromorphic sensing in robotics. *arXiv preprint arXiv:2103.02751*

Hoyer, P. O. (2004). Non-negative matrix factorization with sparseness constraints. *Journal of Machine Learning Research* 5, 1457–1469

Quian Quiroga, R. and Panzeri, S. (2009). Extracting information from neuronal populations: information theory and decoding approaches. *Nature Reviews Neuroscience* 10, 173–185. doi:10.1038/nrn2578

Shannon, C. E. (1948). A Mathematical Theory of Communication. *Bell System Technical Journal* 27, 379–423. doi:10.1002/j.1538-7305.1948.tb01338.x
